# Beyond Flexible: Unveiling the Next Era of Flexible Electronic Systems

**DOI:** 10.1002/adma.202406424

**Published:** 2024-10-11

**Authors:** Min Sung Kim, Amani S. Almuslem, Wedyan Babatain, Rabab R. Bahabry, Uttam K. Das, Nazek El‐Atab, Mohamed Ghoneim, Aftab M. Hussain, Arwa T. Kutbee, Joanna Nassar, Nadeem Qaiser, Jhonathan P. Rojas, Sohail F. Shaikh, Galo A. Torres Sevilla, Muhammad M. Hussain

**Affiliations:** ^1^ mmh Labs (DREAM) Elmore Family School of Electrical and Computer Engineering Purdue University West Lafayette IN 47906 USA; ^2^ Department of Physics College of Science King Faisal University Prince Faisal bin Fahd bin Abdulaziz Street Al‐Ahsa 31982 Saudi Arabia; ^3^ Media Lab Massachusetts Institute of Technology Cambridge MA 02139 USA; ^4^ Department of Physical Sciences College of Science University of Jeddah Jeddah 21589 Saudi Arabia; ^5^ Department of Electrical and Computer Engineering Computer Electrical Mathematical Science and Engineering Division King Abdullah University of Science and Technology (KAUST) Thuwal 23955 Saudi Arabia; ^6^ Logic Technology Development Quality and Reliability Intel Corporation Hillsboro OR 97124 USA; ^7^ International Institute of Information Technology (IIIT) Hyderabad Gachibowli Hyderabad 500 032 India; ^8^ Department of Physics College of Science King AbdulAziz University Jeddah 21589 Saudi Arabia; ^9^ Electrical Engineering Department & Interdisciplinary Research Center for Advanced Materials King Fahd University of Petroleum and Minerals Academic Belt Road Dhahran 31261 Saudi Arabia; ^10^ IMEC Leuven Kapeldreef 75 Leuven 3001 Belgium

**Keywords:** CMOS technology, flexible electronics, transformational electronics

## Abstract

Flexible electronics are integral in numerous domains such as wearables, healthcare, physiological monitoring, human–machine interface, and environmental sensing, owing to their inherent flexibility, stretchability, lightweight construction, and low profile. These systems seamlessly conform to curvilinear surfaces, including skin, organs, plants, robots, and marine species, facilitating optimal contact. This capability enables flexible electronic systems to enhance or even supplant the utilization of cumbersome instrumentation across a broad range of monitoring and actuation tasks. Consequently, significant progress has been realized in the development of flexible electronic systems. This study begins by examining the key components of standalone flexible electronic systems–sensors, front‐end circuitry, data management, power management and actuators. The next section explores different integration strategies for flexible electronic systems as well as their recent advancements. Flexible hybrid electronics, which is currently the most widely used strategy, is first reviewed to assess their characteristics and applications. Subsequently, transformational electronics, which achieves compact and high‐density system integration by leveraging heterogeneous integration of bare‐die components, is highlighted as the next era of flexible electronic systems. Finally, the study concludes by suggesting future research directions and outlining critical considerations and challenges for developing and miniaturizing fully integrated standalone flexible electronic systems.

## Graphical abstract

Flexible electronics are essential in various fields like wearables, healthcare, and environmental sensing due to their flexibility, lightweight construction, and low profile. These systems conform to diverse surfaces, enhancing or replacing bulky instruments in many applications. This study reviews key components of standalone flexible electronics, integration strategies, and recent advancements, emphasizing flexible hybrid and transformational electronics, and concludes with future research directions and challenges.

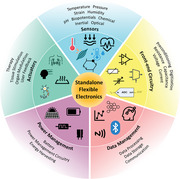

## 1 Introduction

Since its first inception in 1990, flexible and stretchable electronics have garnered significant attention across various domains such as wearables, healthcare, physiological monitoring, human‐machine interface, and environmental sensing.^[^ ^1^ ^]^ Their increasing adoption in many fields is attributed to their excellent flexibility, stretchability, light weight, versatile sensing/actuating capabilities, and fully integrated power management. The distinctive flexibility of these systems is achieved by the utilization of flexible substrates, instead of rigid substrates such as printed circuit boards (PCBs), to host various functional films and inorganic materials. Researchers report various flexible sensors and actuators based on organic materials functionalized with conducting or semiconducting thin films. Lee et al. reported a transparent 2‐µm‐thick bending‐insensitive pressure sensor.^[^ ^2^ ^]^ Liu et al. also reported a simple fabrication of a single‐layer thermocouple on a flexible poly(ethylene terephthalate) (PET) substrate.^[^ ^3^ ^]^ Such flexible sensors exhibit excellent conformity and are optimized to possess high sensitivity. While these sensors can be connected to external analyzers through wires or electromagnetic resonance,^[^ ^4^, ^5^, ^6^ ^]^ their typical usage scenarios often necessitate real‐time processing of sensing parameters in the absence of these external analyzers, requiring the integration of on‐system readout and data management circuits.

**Figure 1:**
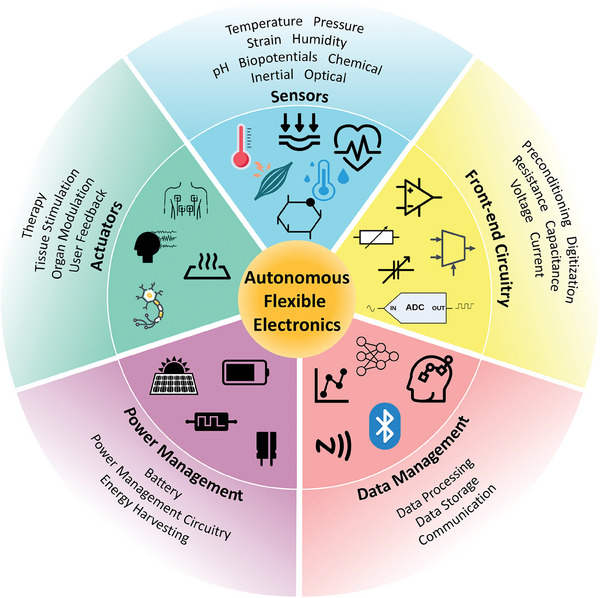
Essential building blocks of fully standalone flexible electronics.

Also, such systems must have a means to transfer the sensed data to external devices for monitoring and feedback. Wireless data transmission, such as through Bluetooth and near‐field communication (NFC), offers alternative communication solutions to such systems, as reported in many studies.^[^ ^7^, ^8^, ^9^, ^10^, ^11^, ^12^, ^13^ ^]^ In addition, for independent operation, they must possess an on‐system power supply, which can take the form of batteries or energy harvesters, and a management circuit. These attributes—sensors, actuators, front‐end circuitry, data management, and power management—constitute the essential building blocks that are indispensable in creating fully standalone flexible electronic systems. **Figure** **1** visually illustrates these fundamental components.

Furthermore, because the vast majority of reported flexible electronics are in the field of wearables and healthcare, they are in direct competition with commercial products such as Apple Watch, Samsung Galaxy Ring, and BioButton by Medtronic with multifaceted functionalities, seamless connectivity, compact integration, and reliable operation.^[^ ^14^, ^15^ ^]^ For instance, the Apple Watch is capable of monitoring skin temperature, ECG, heart rate, and blood oxygen saturation level, detecting fall events and atrial fibrillation, and supporting other useful functions, such as communication and emergency contact service. BioButton offers continuous multimodal non‐invasive sensing of skin temperature, respiratory rate, heart rate, etc.

For flexible electronics to compete with such commercial products in the market, they must possess improved sensing accuracy, versatility, and reliability. Thus, it is imperative to identify the points of improvement and their inherent advantages. The most prominent advantage is flexibility. The application area of commercial products is limited to certain body spots, where the surface is relatively flat, due to their shape and rigidity. For instance, smartwatches can only be worn on the top side of a wrist, and biopatches are typically attached to the chest area. Flexible electronic devices can be attached to surfaces with a smaller curvature due to their inherent flexibility, mitigating the locational limit during deployment.

However, flexibility alone is not a sufficient arsenal, with which flexible electronics can combat commercial products. Thus, the following aspects must be evaluated and improved: miniaturization, high data processing throughput, and full integration.

As for modern electronic systems, flexible electronic systems can also benefit from miniaturization for multiple reasons. First, miniaturization widens the application spectrum. There are many examples of such benefits. Computers went from ENIAC (built in 1991, 1800 square feet) to laptops, which are much smaller and lightweight yet possess better performance granted there is a significant technological gap. Commercial wearable electronics such as the Apple Watch, Samsung Galaxy Ring, and BioButton by Medtronic are all possible due to continuous effort exerted to system miniaturization. In addition, a small system footprint enabled by miniaturization can provide room for additional components for future improvements of an existing system even with extremely stringent area availability.

In the era of increasing data abundance, the capability to capture, store, process, and transmit data in real time with minimal latency is becoming increasingly important. Larger data acquisition, paired with machine learning techniques, enhances the accuracy of physical measurements, especially for wearable systems, whose accuracy is seldom deemed to be worse than that of an implantable system. Wearable systems with highly accurate and precise sensing capabilities can provide non‐invasive alternatives to several invasive solutions, which have limited practicality due to the implantation procedure. To enable such data analysis, not only do flexible electronic systems require application‐specific processing units (e.g., AI accelerators for fast parallel computing), but also technology nodes beyond the ones typically used in the industry for general‐purpose components must be incorporated.

Furthermore, flexible electronic systems are often deployed in remote settings, where they must operate autonomously. As highlighted in the main body of this review, all the essential building blocks—sensors, front‐end circuitry, data management, and power management—must be integrated. Many reported wearable sensing platforms require external analyzers for data acquisition and analysis. Although such strategies are feasible in clinical settings, relying on expensive analyzers prevents the device from reaching a broader group of users. By integrating all the necessary components in the system, the need for such instruments is eliminated and thus the system is rendered more accessible.

Incorporating these qualities, it is possible to construct physically flexible electronic systems with high performance, which are fully integrated in an ultra‐compact manner. Not only can such systems improve existing solutions, but also they can push the envelope by providing novel solutions to hitherto overlooked problems.

There are several reported methods to integrate these building blocks. Organic electronics based on organic substrates and functional materials exhibit excellent flexibility and stretchability and thus are widely investigated for various applications such as e‐skin, human–machine interface, and sensing.^[^ ^16^, ^17^, ^18^, ^19^, ^20^, ^21^ ^]^ Flexible hybrid electronics—systems comprised of flexible substrate and rigid electronic components—show a good compromise between performance and flexibility.^[^ ^7^, ^8^, ^22^, ^23^, ^24^, ^25^ ^]^ Transformational electronics based on the utilization of bare‐die components possess high performance and a potential for miniaturization.^[^ ^26^, ^27^, ^28^, ^29^ ^]^

This review focuses on the essential building blocks and various integration methodologies of flexible electronic systems by examining recent advancements in the field. The first section delves into the essential building blocks of flexible electronic systems. It covers the foundational principles of various sensors, encompassing temperature, pressure, mechanical strain, humidity, pH level, biopotentials, chemical, inertial, and optical sensors, along with their respective sensing modalities. Subsequently, it discusses front‐end circuitry essential for preconditioning, amplifying, and digitizing raw data, as well as methods for processing to derive sensing parameters and transmitting information. Additionally, commonly employed power supply and management strategies are outlined, and actuators utilized for diverse applications such as therapy, stimulation, neuromodulation, and pacemaking are discussed. The subsequent section investigates different approaches for integrating these building blocks to develop fully standalone flexible electronic systems—notably flexible hybrid electronics and transformational electronics—elucidating their recent advancements and distinguishing characteristics. The final section synthesizes crucial aspects pertinent to constructing a fully standalone flexible electronic system and addresses critical considerations and challenges imperative for the continual progression of the field.

## 2 Essential Building Blocks of Flexible Electronic Systems

It is evident that most applications of flexible electronics lie within the field of healthcare and wearables. Therefore, it is of utmost importance for such systems to be fully standalone, that is to have the following components: sensors, sensor‐conditioning circuit, data management (i.e., processing, storage, and transmission), actuation, and power management. In this section, the essential building blocks of fully standalone flexible electronic systems are delineated and discussed from the integration point of view.

### 2.1 Sensors

Similar to how humans perceive the environment and make decisions, sensors inform flexible electronic systems of the surroundings or sensing subject and allow them to actuate and/or communicate with external devices autonomously. Thus, sensors are indispensable parts of flexible electronic systems. Because the majority of applications of flexible electronics is in healthcare and wearables, many sensors in the field are designed to detect physiological parameters and hence infer the physical status of the user. In this section, the materials and mechanisms used to sense various physiological parameters are explored.

#### 2.1.1 Temperature

Temperature sensing is important in monitoring many physiological conditions such as healing processes,^[^ ^37^, ^38^ ^]^ detection of organ transplant rejection,^[^ ^39^ ^]^ skin hydration,^[^ ^40^, ^41^ ^]^ and organ temperature monitoring.^[^ ^42^ ^]^ Considering the compatibility with flexible electronic systems, resistive temperature sensors are popular choices,^[^ ^43^, ^44^, ^45^, ^46^ ^]^ although there are other temperature sensors based on infrared detection^[^ ^47^, ^48^ ^]^ and photoluminescence.^[^ ^49^ ^]^ Resistive temperature sensors rely on the change in electrical resistance in response to the change in the sensor's temperature.

Resistance temperature detectors (RTDs) are made of conductive materials such as gold (Au) and laser‐induced graphene (LIG). RTDs can be used for general temperature sensing because they can be easily fabricated using the standard microfabrication process,^[^ ^39^, ^50^ ^]^ laser ablation^[^ ^51^ ^]^ or direct printing.^[^ ^30^ ^]^ An RTD's resistance changes by the following equation:

(1) $$R_{\mathrm{RTD}}\left(T\right)=R_{\mathrm{ref}}\left(1+\alpha \left(T-T_{\mathrm{ref}}\right)\right)$$

where *R*_RTD_(*T*) is the RTD resistance as a function of temperature (*T*), *R*_ref_ is the resistance of the RTD at a reference temperature (*T*_ref_), and α is the TCR. To enhance the sensitivity of an RTD, it is pivotal to choose a thermally sensitive material. **Table** **1** shows the TCR values of different materials.

**Table 1:** Temperature coefficient of resistance of various materials.^[^ ^10^, ^52^, ^53^, ^54^, ^55^, ^56^ ^]^

Material	α [%K^−1^]	Material	α [%K^−1^]
Silver (Ag)	0.3819	Tungsten (W)	0.4403
Copper (Cu)	0.4041	Iron (Fe)	0.5671
Gold (Au)	0.3715	Platinum (Pt)	0.3729
Aluminum (Al)	0.4308	Nickel (Ni)	0.5866
Carbon (C)	0.115^[^ ^10^ ^]^	Laser‐Induced Graphene (LIG)	−0.44,^[^ ^54^ ^]^ −0.046,^[^ ^52^ ^]^ −0.042,^[^ ^56^ ^]^ −0.04145^[^ ^53^ ^]^
MXene (Ti_3_C_2_T_x_)	−1.07^[^ ^55^ ^]^		

On the other hand, a thermistor is a heterostructure of a thin ceramic layer sandwiched in two metal electrode layers. Thermistors typically have a narrower temperature window (e.g., between −50 and 150 °C) than RTDs (e.g., between −260 and 650 °C).^[^ ^57^ ^]^ Also, unlike RTDs, thermistors have a highly nonlinear temperature response, which can be described using the relationship:^[^ ^58^ ^]^

(2) $$R_{\mathrm{thermistor}}\left(T\right)=R_{\infty }e^{\frac{B}{T}}$$

where *R*_thermistor_(*T*) is the thermistor resistance as a function of temperature, *T* is the temperature, *R*_∞_ is the reference resistance at *T*  =  ∞ K, and *B* is the material constant of thermistor.

**Figure 2:**
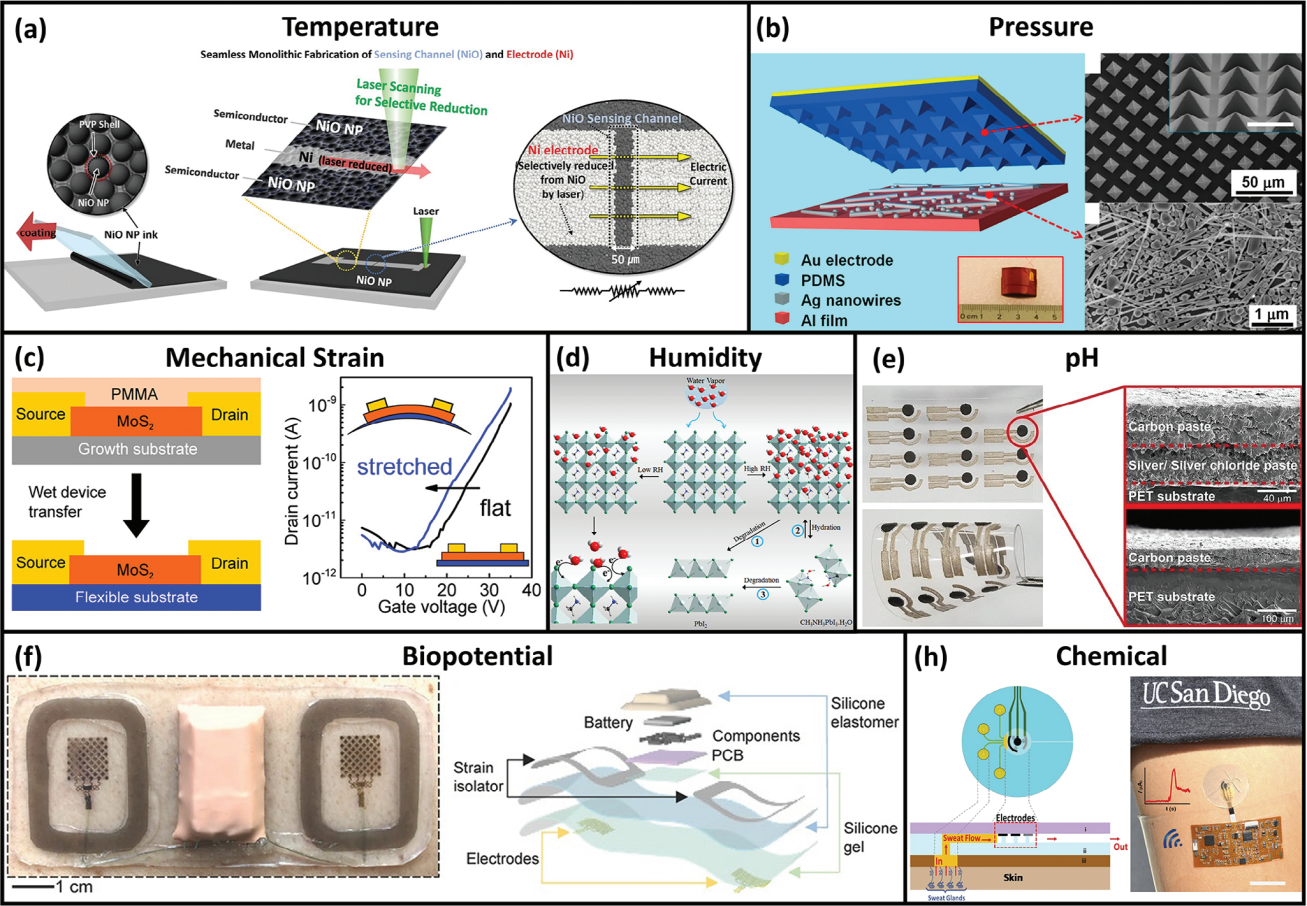
Flexible sensors for various sensing parameters. a) Fabrication process of a flexible temperature sensor based on selective reducing of nickel oxide nanoparticles (NiO NPs) by laser ablation. Reproduced under the terms of the CC‐BY Creative Commons Attribution 4.0 International license.^[^ ^30^ ^]^ Copyright 2019 The Authors, published by Wiley‐VCH. b) Left: 3D illustration of a triboelectric pressure sensor with polydimethylsiloxane (PDMS) micro pyramids and silver nanowires (Ag NWs). Top‐right: Scanning electron microscopy (SEM) image of the PDMS micro pyramids. Bottom‐right: SEM image of Ag NW on an aluminum (Al) film. Reproduced with permission.^[^ ^31^ ^]^ Copyright 2013, American Chemical Society (ACS). c) Left: Transfer printing of molybdenum disulfide (MoS_2_)‐based strain‐sensitive field‐effect transistor (FET). Right: Transfer characteristics of the device with and without stretching. Reproduced with permission.^[^ ^32^ ^]^ Copyright 2015, ACS. d) Electron transfer, hydration and degradation caused by the interaction between perovskite‐based humidity sensing element and moisture. Reproduced with permission.^[^ ^33^ ^]^ Copyright 2019, ACS. e) Optical image of a pH sensor with a carbon working electrode (WE) and a silver/silver chloride (Ag/AgCl) reference electrode (RE) before polyaniline (PANI) polymerization. Insets: Cross‐sectional SEM images of the WE and RE. Reproduced under the terms of the CC‐BY Creative Commons Attribution 4.0 International license.^[^ ^34^ ^]^ Copyright 2019, The Authors, published by Springer Nature. f) Left: Optical image of an electrocardiogram (ECG) sensor with enhanced noise rejection from motion and electromyography (EMG). Right: Exploded view of the device. Reproduced with permission.^[^ ^35^ ^]^ Copyright 2021, Wiley‐VCH Verlag GmbH. g) Left: Cross‐sectional representation of an integrated sweat sensor. Right: Optical image of the device. Reproduced with permission.^[^ ^36^ ^]^ Copyright 2017, ACS.

Thermistors have a faster response time than RTDs, making them suitable for transient thermal analysis.^[^ ^40^, ^41^ ^]^ Shin et al. fabricated a flexible thermistor by selectively reducing NiO NPs using laser ablation (**Figure** **2**a).^[^ ^30^ ^]^ During fabrication, NiO NP ink is uniformly coated on a polyethylene terephthalate (PET) substrate. Two Ni electrodes are defined using laser ablation, with a 50‐µm‐long NiO NP channel—the sensing material—in between. The Ni–NiO–Ni thermistor exhibits a high negative temperature coefficient of resistance of −9.2% K^−1^ in the room temperature range.

#### 2.1.2 Pressure

Pressure is another important sensing parameter in many application areas of flexible electronics. Pressure sensing plays an important role in e‐skin, physiological monitoring, and environmental sensing.^[^ ^59^ ^]^ Pressure can be sensed through different mechanisms including piezoresistance, piezocapacitance, piezoelectricity, triboelectricity, etc. The change in applied pressure modulates the total resistance of the sensing element. The simplicity of piezoresistive pressure sensing makes it attractive for flexible electronic systems, where real estate is limited. The change in resistance can be induced in several different ways. A hybrid composite consisting of an elastomer and liquid metal can be sandwiched between two electrodes.^[^ ^60^ ^]^ The applied pressure changes the number of conductive paths between the electrodes through the sintering process, modulating the total resistance of the composite. The polarity and anisotropy of the piezoresistivity depend on the material and fabrication process.^[^ ^61^, ^62^, ^63^, ^64^ ^]^ Alternatively, conductive NPs can be embedded in an elastomer to achieve a highly sensitive piezoresistive element; in this case, the applied pressure reduces the distance between NPs, thus increasing quantum tunneling and decreasing the resistance.^[^ ^65^, ^66^ ^]^ Another type of piezoresistive pressure sensing is based on very thin homogeneous materials such as silicon (Si) nanomembranes.^[^ ^67^, ^68^, ^69^ ^]^

Piezocapacitive pressure sensing is based on the modulation of capacitance due to the change in applied pressure. Capacitance can be expressed as:

(3) $$C=\varepsilon_{0}\varepsilon_{\mathrm{ins}}\frac{A}{t}$$

where ɛ_0_ is the vacuum permittivity, ɛ_ins_ is the insulator's dielectric constant, *A* is the total capacitor area, and *t* is the distance between the two capacitor plates.

In piezocapacitive pressure sensors, ɛ_ins_ and *t* can be modulated by the applied pressure. In conventional MEMS piezocapacitive pressure sensors, the cavity between the bottom plate and the upper diaphragm can be sealed under vacuum (absolute pressure sensing) or exposed to atmospheric pressure (relative pressure sensing).^[^ ^70^, ^71^ ^]^ A pair of capacitors can be used to achieve differential pressure sensing with enhanced ambient noise rejection. The applied pressure presses down the upper diaphragm, reduces *t* and consequently increases *C*. Similar to MEMS pressure sensors, the modulation of *t* is the most widely used approach in flexible electronics.^[^ ^72^, ^73^, ^74^ ^]^ Instead of air or vacuum with ɛ_ins_  ≈  1, the dielectric material is replaced with elastomers such as PDMS with ɛ_PDMS_  ≈  2.4^[^ ^75^, ^76^ ^]^ and Ecoflex with ɛ_Ecoflex_  ≈  2.17.^[^ ^77^, ^78^ ^]^ Furthermore, by incorporating particles with high ɛ_*ins*_such as liquid metal droplet (LMD),^[^ ^62^, ^79^, ^80^ ^]^ carbon nanotube (CNT),^[^ ^77^, ^78^ ^]^ Ag NPs,^[^ ^81^ ^]^ carbon black particles,^[^ ^82^ ^]^ conducting polymer nanowires,^[^ ^76^ ^]^ it is possible to boost the effective dielectric constant (ɛ_eff_) and thus *C*. Furthermore, by introducing air gaps in the elastomer, ɛ_eff_ can be pressure‐modulated, and thus the sensor's sensitivity can be enhanced.^[^ ^77^, ^81^, ^83^ ^]^

Piezoelectricity is the generation of electrical charge in response to mechanical stress. Typical piezoelectric materials include inorganic materials such as lead zirconate titanate (PZT),^[^ ^84^, ^85^, ^86^ ^]^ barium titanate (BaTiO_3_),^[^ ^87^, ^88^, ^89^, ^90^ ^]^ and organic materials including polyvinylidene fluoride (PVDF).^[^ ^91^, ^92^, ^93^ ^]^ Use of hybrid composites of inorganic and organic materials also has been reported.^[^ ^94^, ^95^ ^]^ Piezoelectric pressure sensing can reduce power consumption and has a good dynamic response.^[^ ^84^, ^86^, ^96^ ^]^ However, this class of pressure sensors suffers from low sensitivity. Triboelectric pressure sensors, which rely on the charge generation of dissimilar triboelectric materials through electrification and electrostatic induction, are alternative solutions to charge‐based pressure sensing.^[^ ^97^, ^98^ ^]^ A notable example is the study by Lin et al. where the upper soft triboelectric layer is patterned in an array of pyramids, which deforms under pressure, resulting in charge generation and thus voltage (see Figure 2b).^[^ ^31^ ^]^ Although both piezoelectric and triboelectric pressure sensing can be made fully flexible and thus compatible with flexible electronic systems, they perform poorly in static sensing due to temporal drift in sensor response.^[^ ^99^ ^]^

#### 2.1.3 Mechanical Strain

The sensing mechanisms of pressure sensing can be also applied to sense mechanical strain. The use of composite materials is more common in piezoresistive strain sensing to ensure the sensor's stretchability and hence detection range. Hybrid materials such as elastomer composites are typically used for this application.^[^ ^100^, ^101^, ^102^, ^103^ ^]^ Sensors based on multilayer materials and in‐situ restructuring also show appreciable piezoresistive properties.^[^ ^104^ ^]^ Piezocapacitive strain sensors can be constructed in several different ways. One way is to simply stack two stretchable capacitor plates so that stretching the capacitor results in an increase in the total capacitor area as well as the distance between the two plates and hence the total capacitance.^[^ ^105^ ^]^ A similar mechanism is used for an interdigitated electrode (IDE) architecture.^[^ ^106^ ^]^ However, if the electrode materials are compliant, repeated stretching will result in microcracks in the capacitor plates over time. To overcome this problem, the capacitor plates can be pre‐stretched before being assembled,^[^ ^107^ ^]^ or stretchable electrode materials such as conductive elastomer composite or liquid metal can be used.^[^ ^108^, ^109^ ^]^ In addition, strain‐sensitive FETs with transition metal dichalcogenides (TMDs) based on bandgap modulation and reduced graphene oxide (rGO) based on interlayer coupling are also reported (Figure 2c).^[^ ^32^, ^110^ ^]^

#### 2.1.4 Humidity

Humidity is an important parameter in physiological monitoring, environmental sensing, agriculture, and e‐skin.^[^ ^111^ ^]^ There are multiple mechanisms that one may take to make a humidity sensor. Notable ones include resistive, capacitive, and impedance‐based sensing. There are other sensing types such as surface acoustic wave (SAW)^[^ ^112^, ^113^, ^114^, ^115^, ^116^ ^]^ and quartz crystal microbalance (QCM).^[^ ^117^, ^118^, ^119^, ^120^, ^121^ ^]^ As mentioned, resistive sensing is widely used for various sensing parameters due to its simplicity, and humidity sensing is not an exception. The absorption and desorption of water molecules into the sensing element changes its resistance. Several material choices including Ag NPs,^[^ ^122^ ^]^ graphene,^[^ ^122^, ^123^ ^]^ CNT,^[^ ^124^ ^]^ and carbon^[^ ^125^ ^]^ have been utilized. The sensitivity varies significantly depending on the material used. Haque et al. have reported the use of hybrid perovskite microstripes, which can quickly absorb water molecules (see Figure 2d).^[^ ^33^ ^]^ The absorbed water molecules enhance the charge carrier transport in the material mainly through proton hopping, reducing the sensor's resistance. The sensor's resistance decreases by four orders of magnitude going from a relative humidity level of 10% to 95%.

Capacitive humidity sensors operate by modulating the dielectric constant of the insulating material responsive to water molecules. Such sensors typically take either a metal–insulator–metal (MIM) structure or an IDE structure. However, unlike resistive sensors, capacitive sensors suffer from slow response and recovery times—the times required for the sensor to respond to water molecules and to stabilize to respond to the next humidity change, respectively—because the humidity‐sensitive dielectric material has only a small contact area with the ambient air. Most capacitive humidity sensors have a slow response/recovery time of several to tens of seconds.^[^ ^126^, ^127^, ^128^, ^129^, ^130^, ^131^, ^132^, ^133^ ^]^

Impedance‐based sensing is another strategy to measure humidity. A typical construction of such a sensor involves an IDE or serpentine electrode with a thin layer of moisture‐sensitive material.^[^ ^134^, ^135^, ^136^ ^]^ The working principle is similar to that of the resistive sensor, but the impedance can be modulated by the operating frequency due to the non‐instantaneous polarization of absorbed water molecules. Impedance‐type sensors provide low hysteresis and high sensitivity.^[^ ^134^ ^]^ However, because at higher frequencies, the impedance drastically drops, a judicious decision must be made when choosing the operating frequency.

#### 2.1.5 pH Level

pH level, a measure of the acidity/alkalinity of a solution, is widely used in sweat sensing,^[^ ^137^, ^138^, ^139^ ^]^ tracking of wound sites,^[^ ^140^ ^]^ agriculture,^[^ ^141^ ^]^ and food quality monitoring.^[^ ^142^ ^]^

(4) $$\mathrm{pH}\;=-\log\left[H^{+}\right]$$

where [H^+^] is the hydrogen ion concentration in the solution.

The majority of pH sensing is done potentiometrically. A potentiometric pH sensor has two electrodes: a reference electrode (RE) and a working electrode (WE). The WE is decorated with pH‐sensitive sensing material to induce a reversible redox reaction between the sensing material and the solution. The redox reaction is based on the protonation and deprotonation of electrochemically active reaction sites on the sensing material. For pH‐sensitive metal oxides such as iridium oxide (IrO_x_) or manganese oxide (MnO_x_), the surface hyrdroxyl groups can be protonated and deprotonated depending on the hydrogen ion concentration. In the case of polyaniline (PANI), which is a widely used conductive polymer as the WE material in pH sensing, the nitrogen atoms in the polymer backbone can accept and release hydrogen ions. Regardless of the sensing material, ideally, the induced potential difference between the WE and RE follows the Nernstian response:^[^ ^143^ ^]^

(5) $$V_{\mathrm{WE}}=V_{\mathrm{WE},0}-2.3\cdot \frac{RT}{nF}\cdot \mathrm{pH}$$

where *V*_WE_ is the induced working electrode potential, *V*_WE,0_ is the standard working electrode potential, *R* is the ideal gas constant, *T* is the temperature, *n* is the number of electrons transferred in the redox reaction, *F* is the Faraday constant, and pH is the pH level.

At room temperature and assuming *n*  =  1, the theoretical maximum limit for pH sensitivity (*S_pH_*), often denoted as the Nernstian limit, is given by:

(6) $$S_{\mathrm{pH}}^{\max}=\frac{\partial V_{\mathrm{WE}}}{\partial \mathrm{pH}}=-2.3\cdot \frac{RT}{F}=-59.2\;\mathrm{mV}\;pH^{-1}$$

In general, the current collector portions of the WE and RE are made of conductive materials such as Au,^[^ ^138^, ^144^, ^145^ ^]^ CNT composites,^[^ ^146^ ^]^ Ag/AgCl,^[^ ^34^, ^147^ ^]^ Pt,^[^ ^148^, ^149^ ^]^ carbon paste,^[^ ^140^ ^]^ and graphite.^[^ ^139^ ^]^ The current collectors are decorated with different functional materials. In many studies, Ag/AgCl RE has been utilized.^[^ ^138^, ^139^, ^140^, ^145^, ^146^, ^147^, ^148^, ^149^, ^150^, ^151^, ^152^ ^]^ The main attributes that led to its widespread adoption as the RE material include minimal medium contamination, facile preparation, potential for miniaturization, and independence of electrode orientation.^[^ ^153^ ^]^ Although Ag/AgCl RE exhibits stable electrical potential, if exposed to low‐conductivity or reductive solution for a long time, the AgCl gets dissolved into the solution as silver complex anions.^[^ ^154^ ^]^ This consequently imperils the electrical potential stability of the RE.

On the other hand, many studies have reported various functional materials for the WE. Although most sensors exhibit sub‐Nernstian pH sensitivity,^[^ ^140^, ^142^, ^145^ ^]^ super‐Nernstian pH sensitivity values have been achieved by coating the WE with IrO_x_,^[^ ^139^, ^148^, ^149^ ^]^ defect‐engineered graphene^[^ ^155^ ^]^ and MnO_x_.^[^ ^156^ ^]^ Other WE materials include PANI,^[^ ^34^, ^137^, ^138^ ^]^ polyacrylic acid‐CNT,^[^ ^146^ ^]^ and graphite‐polyurethane (see Figure 2e).^[^ ^144^, ^147^ ^]^ It should be noted that the WE material choice and fabrication method greatly affect the WE's pH sensitivity. In addition, for general pH sensing, PANI is widely used to coat the WE due to its facile polymerization process and high sensitivity towards hydrogen ions.^[^ ^34^, ^157^, ^158^, ^159^ ^]^

The advantage of potentiometric pH sensing is that it can be easily interfaced with microcontroller units (MCUs) via an amplifying circuit and an analog‐to‐digital converter (ADC). Furthermore, such pH sensors can be embedded in a microfluidics channel/reservoir, which may hold other chemical sensors for electrolytes, metabolites, drugs, etc.^[^ ^10^, ^137^, ^157^ ^]^ A more thorough review of electrochemical pH sensing can be found in ref. [160].

#### 2.1.6 Biopotentials

Biopotentials are bioelectrical signals vital in the physiological monitoring of various parts of the human body. They include electrocardiogram (ECG), electromyogram (EMG), electroencephalogram (EEG), and electrooculogram (EOG), which originate from the heart, muscle, brain, and eyes, respectively, in the form of electrical potential.

In the heart, potential impulses are spontaneously generated by the sinoatrial (SA) node and subsequently propagated to the atrioventricular (AV) node, where the impulses are held to create a delay for the atrial muscle to relax. When the atrial muscle is relaxed, the AV node releases the impulses through the His and Purkinje fibers to the ventricular muscle, which contracts in response to the received impulses and pumps out blood. ECG encodes the pumping cycle in the form of electrical potential, which has different artifacts representing different phases within a cycle. Thus, by analyzing ECG signals, it is possible to diagnose the heart's conditions and detect any abnormalities, which makes it a primary diagnostic tool for cardiovascular diseases.^[^ ^161^, ^162^ ^]^ In addition to diagnostics, it can be used to monitor patients during surgery, medication, and recovery.^[^ ^163^ ^]^ ECG can also be used to monitor an athlete's conditions for performance optimization^[^ ^164^, ^165^ ^]^ and continuous daily monitoring (Figure 2g).^[^ ^35^ ^]^

EMG, on the other hand, encodes the electrical muscular activities. The electrical motor potential originates from the motor cortex in the brain and gets transmitted through the central nervous system to the destination muscle. The potential acts like a nerve stimulation, which causes the muscle to contract. Unlike ECG which measures repetitive involuntary control of the heart muscle, EMG signals encode the voluntary intention for peripheral muscle contraction.^[^ ^166^ ^]^ This makes EMG suitable for many motor‐related applications such as prosthetics control,^[^ ^167^, ^168^, ^169^, ^170^ ^]^ post‐stroke rehabilitation,^[^ ^171^, ^172^ ^]^ and neuromotor disorders.^[^ ^173^ ^]^

As a non‐invasive alternative to electrocorticogram (ECoG), which is directly measured from the cortical surface of the brain, EEG can be measured on the scalp or forehead to probe the electrical activity generated by the synchronized activity of neurons in the brain.^[^ ^174^, ^175^ ^]^ There are five different EEG spectral waves, each associated with a different task/stimulus and frequency. For instance, θ wave is associated with drowsiness and frequency between 4 and 8 Hz, while γ wave is associated with concentration and frequency between 30 and 44 Hz.^[^ ^176^ ^]^ Proper filtering and spectral decomposition of measured EEG can be used for sleep monitoring,^[^ ^175^, ^177^, ^178^ ^]^ seizure prognosis,^[^ ^179^ ^]^ epilepsy diagnosis/monitoring,^[^ ^180^, ^181^ ^]^ and human–machine interface.^[^ ^182^, ^183^, ^184^, ^185^ ^]^

Similar to EMG, EOG records the electrical activities of the muscles surrounding the eyes. Ocular movements (e.g., blinking, saccadic, smooth pursuit, vergence, and vestibulo‐ocular movements) generate an electrical potential difference between the cornea and retina, which can be detected by placing electrodes around the eyes.^[^ ^186^, ^187^ ^]^ EOG's ability to encode eye movements has many applications in sleep monitoring,^[^ ^175^, ^178^, ^188^ ^]^ assistive technology,^[^ ^189^ ^]^ eye tracking for virtual reality (VR),^[^ ^190^, ^191^ ^]^ and human‐machine interface.^[^ ^192^, ^193^ ^]^

Measurement methods for biopotentials can be done invasively or non‐invasively.^[^ ^194^, ^195^, ^196^ ^]^ Invasive biopotential measurements are done with implantable electrodes, which are inserted into the biological tissue or subdermally implanted. Implantable electrodes generally offer higher signal accuracy and lower noise or interference and can be used to acquire ECG,^[^ ^197^, ^198^, ^199^ ^]^ ECoG,^[^ ^200^, ^201^ ^]^ and EMG.^[^ ^202^ ^]^ Despite their data fidelity, implantable sensors are difficult to use in home settings and cause discomfort in patients/users. Transient implantable monitoring devices based on bioresorbable polymers and metals have been reported,^[^ ^203^ ^]^ but the implantation of these devices still requires surgery. Thus, to maximize the application spectrum, non‐invasive wearable electrodes are more widely integrated with flexible electronic systems. Wearable electrodes are interfaced with the skin at various locations specific to each biopotential type. They can categorized into two: wet and dry. Traditionally, Ag/AgCl wet electrodes are used with hydrogel to enhance the electrodes’ conductivity.^[^ ^163^ ^]^ However, hydrogels dry over time, causing contact issues, and may cause discomfort and skin irritation. Thus, dry electrodes are the preferred choice in miniaturized flexible electronic systems intended for long‐term continuous biopotential monitoring. Au is typically used material for dry electrodes.^[^ ^35^, ^170^, ^204^, ^205^, ^206^, ^207^ ^]^ For high data fidelity, it is important to maintain good contact between the electrode and the skin. Different electrode designs have been reported including strain isolation and polymer‐grafted electrode layer to overcome the mechanical mismatch and improve the electrode interface with the skin.^[^ ^35^, ^208^ ^]^ In addition to direct contact, ECG can be measured capacitively, which allows the ECG measurement over clothes.^[^ ^209^, ^210^, ^211^ ^]^ However, when used over clothes, the capacitance variation caused by the continuously changing gap between the skin and electrode should be compensated for reliable measurement.

#### 2.1.7 Chemical

Bio‐analytes such as metabolites and electrolytes in body fluids are garnering attention in physiological monitoring. Typical body fluids in consideration include blood, sweat, saliva, tears, interstitial fluids, and urine.^[^ ^212^, ^213^ ^]^ Yet, not all body fluids are compatible with non‐invasive wearable solutions. Blood and interstitial fluids are inside of the human body and therefore require techniques such as venipuncture and microneedles, which are painful and invasive.^[^ ^214^, ^215^, ^216^ ^]^ Tear sensing poses safety risks and irritates the eyes. This causes the tear glands to produce reflex tears, which may alter the analyte concentration and hence the sensor reading.^[^ ^217^ ^]^ Continuous monitoring of the analytes in urine is also challenging due to the difficulties lying in the collection. Amongst all body fluids, sweat sensing has attracted much attention due to the rich information available in sweat analytes including metabolites and electrolytes.^[^ ^212^, ^218^ ^]^ Metabolites such as lactate, glucose, and uric acid are typically measured by amperometry, and electrolytes such as sodium (Na^+^), chloride (Cl^−^), potassium (K^+^), and ammonium (NH_4_^+^) are measured using potentiometry as well as amperometry.

Similar to pH sensing, many electrochemical sensors utilize RE made of Ag/AgCl.^[^ ^219^, ^220^, ^221^, ^222^, ^223^, ^224^, ^225^, ^226^, ^227^ ^]^ To decorate the WE, enzymes, notably oxidases, are widely used in metabolite sensing. Oxidases oxidize metabolites and produce hydrogen peroxide (H_2_O_2_) as the reaction byproduct. Examples of glucose, lactate, and uric acid are given below:

(7) $$\mathrm{Glucose}+H_{2}O+O_{2}\overset{\mathrm{GOx}}{\to }\mathrm{Gluconic}\;\mathrm{Acid}+H_{2}O_{2}$$

(8) $$\mathrm{Lactate}+H_{2}O+O_{2}\overset{\mathrm{LOx}}{\to }\mathrm{Pyruvate}+H_{2}O_{2}$$

(9) $$\mathrm{Uric}\;\mathrm{Acid}+H_{2}O+O_{2}\overset{\mathrm{UOx}}{\to }\mathrm{Allantoin}+CO_{2}+H_{2}O_{2}$$

GOx, LOx, and UOx correspond to glucose, lactate, and urate oxidases, respectively.

To induce an amperometric response from H_2_O_2_, Prussian blue (PB or Fe_4_^III^[Fe^II^(CN)_6_]_3_) is often incorporated underneath the enzymatic layer. PB acts as a catalyst in the following redox process:^[^ ^228^ ^]^

(10) $$H_{2}O_{2}+2e^{-}\to 2OH^{-}$$

The redox reaction reduces the H_2_O_2_ into two hydroxide ions (OH^−^) and oxidizes the PB by transferring two electrons from the reduced PB—also known as Prussian white (PW)—to the H_2_O_2_, forming PB. The PB can be reduced back to PW by transferring two electrons from the underlying conductive current collector to the PB. To induce the redox reaction and thus a current flow through the WE, a constant potential should be applied between the WE and RE. However, the reaction at the WE shifts the applied potential. To avoid the potential shift, a third electrode named the counter electrode (CE) lets the current flow between the WE and CE instead. CEs are much larger than the other two electrodes to circumvent current limiting. Also, they are made of inert materials such as Au,^[^ ^222^, ^226^ ^]^ Ag/AgCl,^[^ ^227^ ^]^ and carbon‐based materials^[^ ^219^, ^220^, ^221^, ^223^, ^225^ ^]^ to prevent chemical reactions and hence avoid altering the electrochemical reaction of the WE. The current through the CE then can be measured for amperometric analysis.

Although sensing materials such as oxidases and ionophores are widely used for bio‐analyte sensing, such organic materials come with long‐term stability issues. For instance, in the case of oxidase‐based WEs, OH^−^ can break the Fe^II^‐(CN)‐Fe^II^ bond and produce soluble ferrocyanides ([Fe^II^(CN)_6_]^4−^) through hydrolysis:^[^ ^229^ ^]^

(11) $${\mathrm{Fe}}_{4}^{\mathrm{III}}{\left[Fe^{\mathrm{II}}{\left(\mathrm{CN}\right)}_{6}\right]}_{3}+12OH^{-}\to 4\mathrm{Fe}{\left(\mathrm{OH}\right)}_{3}+3{\left[Fe^{\mathrm{II}}{\left(\mathrm{CN}\right)}_{6}\right]}^{4-}$$

This process gradually decomposes PB. To combat this, materials exhibiting better inertness than PB or nonenzymatic sensing materials have been utilized as the transduction layer.^[^ ^10^, ^221^, ^224^, ^226^, ^230^ ^]^

As opposed to metabolites, electrolytes such as Na^+^, Cl^−^, K^+^, and NH^4+^ are measured with two‐electrode potentiometry. The RE is made of Ag/AgCl, similar to pH and metabolites. Conventionally, a mixture (or cocktail) of ion‐specific ionophores, polymers and solvent is dropcasted to transform the WE to an ion‐selective electrode (ISE).^[^ ^10^, ^231^, ^232^ ^]^ An ionophore is a chemical species that can reversibly bind specific ions; this allows ionophores to capture ions and transport them across the membrane of a biological cell.^[^ ^233^ ^]^ It is possible to replicate the ion‐specific membrane of a biological cell and implement it on an electrode to fabricate an ion‐selective WE. Although organic ion‐selective materials are predominantly used in electrolyte sensing, inorganic materials such as Na_0.44_MnO_2_ can be also used to detect ions.^[^ ^234^ ^]^ Ion‐selective materials are able to go through redox reaction with the specific ion.

In general, multiple analyte‐sensing electrodes are integrated into a single microfluidic reservoir, where sweat collected by iontophoresis is contained until the measurement is done.^[^ ^10^, ^55^, ^137^, ^235^ ^]^ Saliva is another type of body fluid that can be integrated into flexible electronic systems as it also contains various metabolites and electrolytes.^[^ ^236^, ^237^ ^]^ Glucose sensor is the most widely used application of salivary sensing, as it is correlated to the blood glucose level.^[^ ^238^, ^239^, ^240^, ^241^ ^]^ Although the working principles of salivary sensors are similar to those of sweat sensors, salivary sensors have stricter integration requirements since the available real estate is smaller. **Table** **2** summarizes the recognition materials and electrochemical sensing mechanisms of different bio‐analytes.

**Table 2:** In vivo sensing mechanisms and working electrode (WE) materials of various bio‐analytes.

Analyte type	Analyte name	In vivo sensing mechanism	WE material	Refs.
Metabolites	Glucose	Amperometry	Glucose oxidase (GO_x_)	[10, 55, 222, 225, 230, 235]
		Chronoamperometry		[219, 226]
		Cyclic voltammetry (CV)		[242]
	Lactate	Amperometry	Lactate oxidase (LO_x_)	[10, 220, 223, 230, 235]
		Chronoamperometry		[243]
	Uric acid	Amperometry	Urate oxidase (UO_x_)	[10, 235]
		Chronoamperometry		[219, 227]
		Differential pulse voltammetry (DPV)	Urate oxidase (UO_x_)	[244]
			N‐doped reduced graphene oxide	[224]
Electrolytes	Sodium (Na^+^)	Potentiometry	Na_0.44_MnO_2_	[234, 245]
			ISM (0.7 wt.% Na ionophore X, 0.2 wt.% KTCPB, 33 wt.% PVC and 66.1 wt.% NPOE)	[231]
			ISM (1 mg Na ionophore X, 0.55 mg KTCPB, 30 mg PVC, 30 mg SEBS and 65 mg DOS)	[10]
	Chloride (Cl^−^)	Potentiometry	Ag/AgCl	[246, 247, 248]
	Potassium (K^+^)	Potentiometry	K_2_Co[Fe(CN)_6_]	[245]
			ISM (10 wt.% valinomycin and 5 wt.% KTCPB)	[232]
			ISM (2 mg valinomycin, 0.5 mg NaB(C_6_H_5_)_4_, 30 mg PVC, 25 mg SEBS and 70 mg DOS)	[10]
	Ammonium (NH_4_^+^)	Potentiometry	InVO_4_	[249]
			CuHCF	[250]
			ISM (1 mg nonactin, 30 mg PVC, 30 mg SEBS and 65 mg DOS)	[10]

Abbreviations: DOS, bis(2‐ethylhexyl) sebacate; ISM, ion‐selective membrane; KTCPB, potassium tetrakis(4‐chlorophenyl)borate; NPOE, nitrophenyl octyl ether; PVC, polyvinyl chloride; SEBS, styrene‐ethylene‐butylene‐styrene.

#### 2.1.8 Other Sensing Mechanisms

Apart from the so far mentioned sensor types, other sensors can useful insights into the physiological state of the user. Acceleration measurements can provide a wealth of information about the user's conditions depending on where the sensor is located. The most basic use of an acceleration sensor is movement tracking, which has applications in fall detection^[^ ^251^ ^]^ and activity monitoring.^[^ ^252^, ^253^, ^254^ ^]^ In addition, accelerometers can be used to estimate other physiological parameters including seismocardiogram (SCG)—mechanical wave produced by the heart's movement—and respiration rate.^[^ ^9^, ^205^, ^206^, ^252^ ^]^ Many flexible hybrid electronic systems feature the use of rigid surface‐mount device (SMD) accelerometers.^[^ ^9^, ^205^, ^206^, ^251^, ^252^, ^253^, ^254^ ^]^ Some researchers have reported MEMS accelerometers with a rigid proof mass on flexible substrates such as polyimide (PI).^[^ ^255^, ^256^ ^]^ For improved mechanical reliability, flexible accelerometers with soft liquid metal droplet proof mass have been reported as well.^[^ ^257^, ^258^ ^]^ Also, SMD light‐emitting diodes (LEDs) and photodiodes (PDs) are used to measure the photoplethysmogram (PPG), which gives insights into the oxygen saturation in blood and tissue.^[^ ^252^, ^254^, ^259^ ^]^

### 2.2 Data Management

**Figure 3:**
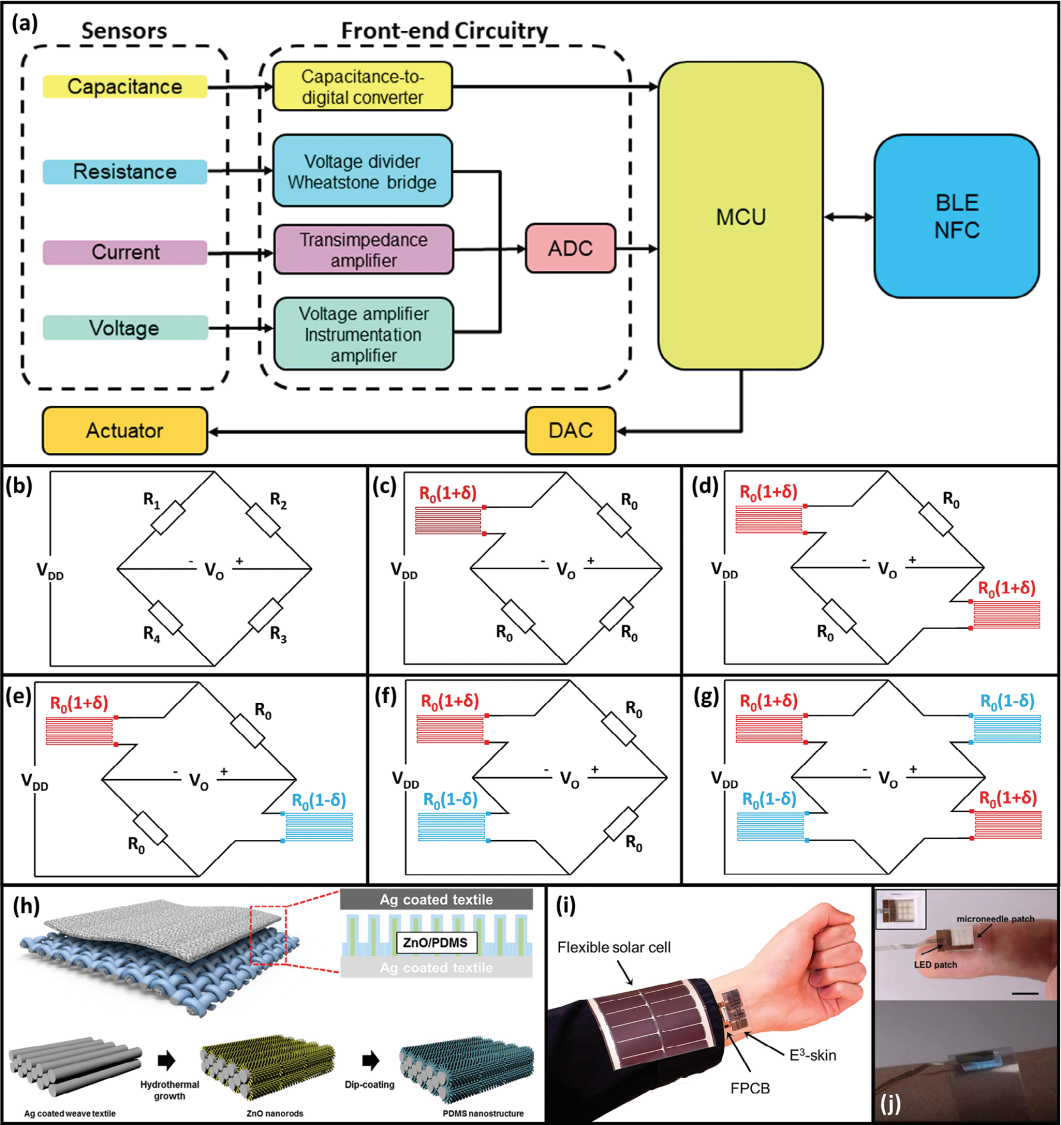
a) Illustration of data flow between various sensors, an MCU, a BLE module, and an actuator. Abbreviations: BLE, Bluetooth Low Energy; DAC, digital‐to‐analog converter. b) Wheatstone bridge circuit. c–g) Wheatstone bridge circuits with various configurations shown in Table 3. h) Top: 3D model of a textile‐based triboelectric nanogenerator. Inset: Device cross‐section. Bottom: Steps for the decoration of an Ag‐coated textile with zinc oxide (ZnO) nanorods and PDMS for the device bottom layer. Reproduced with permission.^[^ ^260^ ^]^ Copyright 2015, ACS. i) Optical image of an integrated physiological sensing platform with a flexible solar cell and supercapacitor for temporary energy storage. Reproduced under the terms of the CC‐BY Creative Commons Attribution 4.0 International license.^[^ ^55^ ^]^ Copyright 2023, The Authors, some rights reserved; exclusive licensee American Association for the Advancement of Science (AAAS), published by AAAS. j) Optical images of PDMS microneedle light guide for deep‐tissue UV light delivery. Reproduced with permission.^[^ ^261^ ^]^ Copyright 2021, Wiley‐VCH.

Output data produced by the various sensor types mentioned in Section 2.1 must be properly handled to enable reliable and continuous monitoring of sensing parameters. Data management can be largely divided into two stages: front‐end circuit and data processing/transmission. **Figure** **3**a illustrates the data flow for a typical flexible electronic system.

#### 2.2.1 Front‐End Circuit

As shown in Section 2.1, there is a myriad of sensors, each of which can be detected using different sensing mechanisms including resistance, capacitance, voltage, and current. In fully standalone systems, MCUs or other control logic units are integrated to perform data processing. Because MCUs are not capable of directly detecting electrical parameters other than voltage, if the sensor output is not in the form of voltage, it must be converted into a voltage first by an analog front‐end circuit. Then, the converted voltage is amplified (and filtered if necessary), so that it is in the range detected by the MCU. It is a common practice to combine conversion and amplification into the analog front‐end circuit.

For a resistive sensor, the change in the sensing parameter induces a change in its resistance. Circuits used to convert the resistance change into a voltage include a voltage divider, the Wheatstone bridge,^[^ ^262^, ^263^, ^264^ ^]^ time‐domain readout,^[^ ^263^, ^264^, ^265^, ^266^, ^267^, ^268^, ^269^, ^270^, ^271^ ^]^ and a direct resistor‐to‐MCU interface.^[^ ^272^, ^273^ ^]^ Voltage dividers are widely used as the interface circuitry for resistive sensors, especially temperature sensors, due to their implementation simplicity and small footprint. However, because the voltage divider constantly draws current from the power supply, for low‐power applications, the resistance values of the resistive sensing element and resistor should be moderately large.

Wheatstone bridge circuits generate output voltage based on the resistance imbalance caused by the change in resistance of the resistive sensor (see Figure 3b). When the resistor network is balanced, the output voltage is zero. If the resistance of the resistive sensor changes due to a change in the sensing parameter, there will be a resistance imbalance, and the output voltage will increase. This output voltage typically does not require amplification and can be quantized using an ADC. There are several Wheatstone bridge configurations such as quarter‐bridge, half‐bridge and full‐bridge, each with different output voltage relationships. **Table** **3** shows the output voltage of different Wheatstone bridge configurations shown in Figures 3c–g.

**Table 3:** Output voltages (*V*_o_) of different Wheatstone bridge configurations shown in Figure 3a. Corresponding circuit diagrams are shown in Figure 3c–g.

Figure No.	Type	*R*_1_	*R*_2_	*R*_3_	*R*_4_	*V_O_*
3c	Quarter‐bridge	*R*_0_(1 + δ)	*R*_0_	*R*_0_	*R*_0_	$V_{DD}\frac{\delta }{4+2\delta }\approx V_{DD}\frac{\delta }{4}$
3d	Half‐bridge	*R*_0_(1 + δ)	*R*_0_	*R*_0_(1 + δ)	*R*_0_	$V_{DD}\frac{\delta }{2+\delta }\approx V_{DD}\frac{\delta }{2}$
3e		*R*_0_(1 + δ)	*R*_0_	*R*_0_(1 − δ)	*R*_0_	$-V_{DD}\frac{\delta^{2}}{4-\delta^{2}}\approx -V_{DD}\frac{\delta^{2}}{4}$
3f		*R*_0_(1 + δ)	*R*_0_	*R*_0_	*R*_0_(1 − δ)	$V_{DD}\frac{\delta }{2}$
3g	Full‐bridge	*R*_0_(1 + δ)	*R*_0_(1 − δ)	*R*_0_(1 + δ)	*R*_0_(1 − δ)	*V_DD_* δ

*R*_0_ is the nominal resistance, and δ is the fractional change in resistance (= Δ*R*/*R*_0_ ). *V*_DD_ is the supply voltage. Approximations are given assuming δ ≪ 1.

However, Wheatstone bridges possess several limitations. First, similar to a voltage divider, the circuit constantly draws power, which can be disadvantageous in low‐power applications. Also, zero drift error—a non‐zero output voltage when there is no change in the resistive sensor's resistance—can be caused by mismatches in the components and changes in the ambient conditions during operation (e.g., temperature).^[^ ^274^ ^]^ This reduces the sensing range and shift in the sensor reading. Furthermore, if the Wheatstone bridge is of push‐pull type, the output voltage will exhibit nonlinearity, which jeopardizes the sensor's accuracy.^[^ ^275^, ^276^, ^277^ ^]^ For example, a push‐pull Wheatstone bridge involves a pair or two of two temperature sensors with positive and negative temperature coefficients or resistance (TCRs). Moreover, because the output voltage is susceptible to interference and power supply noise, without proper filters for noise rejection and a stable power supply, the accuracy of the sensor can be compromised.

To enhance the immunity against noise and the linearity of the readout circuit, many have adopted the use of time‐modulated digital output instead of directly quantizing the analog output voltage to infer the change in sensing parameters.^[^ ^263^, ^264^, ^265^, ^266^, ^267^, ^268^, ^269^, ^270^, ^271^ ^]^ Such a linear digitizer contains an oscillator, often consisting of an integrator and a comparator. The oscillator produces a square wave at the out, whose period is modulated by the change in the resistance of the resistive sensor. There are different configurations as to how the resistive sensor can be incorporated into the circuit; it can be a part of a Wheatstone bridge^[^ ^263^, ^264^, ^270^ ^]^ or a current mirror/source.^[^ ^265^, ^266^ ^]^ Some have integrated the sensor directly into the integrator.^[^ ^267^, ^268^, ^269^, ^271^ ^]^ Finally, the period‐modulated square wave can be fed into a GPIO pin of the MCU to infer the change in the sensing parameter; each period value corresponds to a resistance value, which is related to a specific sensing parameter value. In addition to the improved noise immunity and linearity, time‐domain readout circuits possess a higher resolution and larger dynamic range. Moreover, because the output of such a readout circuit is a binary square wave, it does not need to be interfaced with an ADC. However, compared to simple Wheatstone bridge circuits, time‐domain readout circuits require more passive and active components. Not only does it increase the overall circuit complexity, but also it increases the power consumption and area of the system. In flexible electronics, because the power supply is limited and system footprint must be minimized, unless application‐specific integrated circuits (ASICs) are utilized, such complex readout circuitry is not typically used.

Similar to resistive readout circuits, capacitive readout circuits are designed to convert the change in the capacitive sensor's capacitance into an analog voltage. Depending on the sensor type, the capacitive front‐end circuit may be non‐differential (e.g., pressure sensor) or differential (e.g., accelerometer). Regardless of the circuit type, the capacitance‐to‐voltage conversion involves operational amplifiers and clocked switches to perform the conversion and appropriate amplification. In the case of non‐differential circuits, a reference capacitor is needed to detect the change in capacitance.^[^ ^278^, ^279^, ^280^, ^281^, ^282^, ^283^, ^284^, ^285^, ^286^ ^]^ On the other hand, differential circuits rely on the capacitance mismatch to induce a charge imbalance.^[^ ^287^, ^288^, ^289^, ^290^, ^291^, ^292^, ^293^, ^294^ ^]^ Since the exact working principle of a capacitive readout circuit substantially differs from circuit to circuit, it is not further discussed in this review. Readers are encouraged to refer to ^[^ ^286^, ^295^ ^]^.

However, in practice, due to the complexity of implementing a capacitive readout circuit from the ground up, many flexible electronic systems utilize commercial off‐the‐shelf (COTS) capacitance‐to‐digital converters (CDCs) to facilitate the integration process and ensure reliable data conversion. COTS CDCs such as FDC1004 by Texas Instruments, AD7745 by Analog Devices are integrated with built‐in ADCs supporting I^2^C serial interface, which can be directly interfaced to an MCU for subsequent data analysis and transmission.

For potentiometric sensors, no special preconditioning circuit is required other than a voltage amplifier because the voltage variation caused by potentiometric sensors (e.g., electrochemical) is typically tens of mV. An amperometric sensor, which converts variations in the sensed parameter into an electrical current, needs a trans‐impedance amplifier to transform the current into a voltage. Due to the fact that amperometric electrochemical sensors produce output current densities ranging from several to tens of µA cm^−2^, it is essential for the trans‐impedance amplifier to possess a high gain to ensure reliable current detection.

After the preconditioning, the converted analog voltage must be digitized before being fed into the MCU for further processing. The two largely investigated groups of ADCs include successive approximation register (SAR) and sigma‐delta (ΣΔ) modulation. The former operates by sequentially approximating the input analog signal from the most significant bit (MSB) to the least significant bit (LSB). The conversion done by a SAR ADC is fast and power‐efficient but can suffer in high‐precision applications due to limited bit resolution. On the other hand, ΣΔ ADCs can attain a higher resolution by oversampling the input analog signal at a frequency much higher than that of the input signal followed by a decimation filter (e.g., downsampling). However, it comes at the cost of increased power consumption due to higher operating frequency and digital filtering. In flexible electronic systems, the power supply's capacity is typically limited, and the incorporated sensor may demand a high‐resolution readout circuit, necessitating a performance‐energy co‐optimization.

#### 2.2.2 Data Processing/Transmission

The data acquired by the sensor and subsequently preconditioned by the front‐end circuit should then be further processed to infer the change in the sensing parameters discussed in Section 2.1. A calibration step is indispensable because it can empirically confirm the relationship between the digital data and the change in the sensing parameter. It can also account for any nonlinearity present in the sensor. A reference high‐precision system is often used to independently measure the sensing parameter along with the sensor to be calibrated. An empirical relationship can be derived from this process and can be programmed into the system's MCU to take the measured data as the input and back‐calculate the sensing parameter as the output.

If there is no simple empirical relationship between the data and the sensing parameter, more sophisticated approaches including filtering, support vector machines (SVMs), regression models, and neural networks can be used to extract the sensing parameter from the raw data. However, not only do conventional machine learning (ML) algorithms demand high computational load and thus consume more power, but also many general‐purpose MCUs lack the computational power, unable to perform such tasks, necessitating the use of a computing module with parallel computing capabilities. If so, the digitized raw data can be directly transmitted to an external device with superior computational power (e.g., smartphone or tablet), so that it can analyze the raw data and extract the sensing parameters. Examples include inferring the physiological status of an individual using biopotentials,^[^ ^203^, ^206^, ^296^ ^]^ acceleration,^[^ ^9^ ^]^ and temperature.^[^ ^40^ ^]^

In healthcare and wearables, systems are generally deployed to monitor the status of the user by sensing physiological data (e.g., temperature, biopotentials). If the sole purpose of collecting the physiological data is to autonomously control the on‐system actuators such as electrical stimulation^[^ ^297^ ^]^ and phototherapy modules,^[^ ^261^ ^]^ communication with external devices is not necessary. However, if the sensor data should be transmitted to an external device to inform the user of the acquired data, the system must possess communication capabilities, which can be wired or wireless. Wired communication can establish a robust connection between the flexible system and the external server. A wired connection can also allow direct usage of an external precision analyzer to analyze the sensed data. However, if wired communication is used, the distance between the flexible electronic system and the external analyzer is strictly limited to the length of the wire, forcing the two systems to be in close proximity.^[^ ^170^, ^298^ ^]^ Furthermore, for wearable applications, a wired connection makes the system bulkier and thus causes discomfort to the user.

Thus, wireless communication is the preferred method and is commonly used in the field.^[^ ^10^, ^299^, ^300^ ^]^ There are several wireless communication schemes including near‐field communication (NFC) and Bluetooth. NFC works by a master device generating an electromagnetic field at a radio frequency of 13.56 MHz and inductively coupling the slave device for communication.^[^ ^301^, ^302^ ^]^ It has a data transfer rate between 0.02 and 0.4 Mbps and a communication range of 4–10 cm, which limits its application spectrum. Because of this, NFC technology is often with the so‐called “touching paradigm,” where the active master device comes in close contact with the passive (or possibly active) slave device for communication.^[^ ^303^ ^]^ Despite its short communication range, NFC is useful when range is not a critical factor. It is also favorable if there is no on‐system power supply. For instance, Choi et al. reported a passive, sensor‐loaded implant device for monitoring and electrical stimulation.^[^ ^203^ ^]^ A complementary skin‐mounted wearable system with a lithium polymer battery communicates with and powers the implant device through NFC to ensure the transient nature of the implant and eliminate the need for additional surgery to remove the implant. However, a long‐range communication scheme was still used to enable data communication between the on‐skin wearable assembly and a smartphone.

The most commonly used long‐ranged communication protocol is BLE.^[^ ^38^, ^56^, ^251^, ^252^, ^299^, ^304^, ^305^ ^]^ BLE modules operate at 2.4 GHz for communication, and depending on the manufacturer and model they consume ≈3–9.7 mA and 5.2–13 mA of peak current while receiving and transmitting data, respectively; in idle mode, they consume 1–2.6 µA of current.^[^ ^306^, ^307^ ^]^ By switching between different modes (e.g., idle, advertising, and connection modes) and modulating the connection interval, BLE modules can simultaneously achieve low power consumption and high data rate, which can be as high as 1–2 Mbps. Furthermore, many BLE modules come in the form of a system‐on‐chip (SoC) with an internal MCU, memory, and ADCs. This decreases the number of components to be integrated, thereby reducing the overall footprint of the final system.

Although wireless transmission of raw data to an external device for analysis can be a solution, this leaves the deployed flexible electronic system incognitive. Edge computing can be implemented in resource‐limited hardware such as MCUs.^[^ ^308^, ^309^ ^]^ Utilizing edge computing reduces the latency between data collection and analysis. For instance, in healthcare, edge‐computing‐enabled artificial intelligence (AI) can augment flexible electronic systems with the ability to extract essential information from the sensed physiological data and autonomously adjust the therapeutic regimens without the invention within the boundary set by medical professionals, thus facilitating the patient's treatment.^[^ ^310^ ^]^ Conventional edge computing strategies face several challenges including difficulties in on‐site ML model retraining, and developing a standardized protocol due to hardware‐dependent processor architecture and hardware limitations. TinyML, which is a framework capable of performing on‐site data analysis with mW or below power consumption, is gaining traction in the IoT field. Although currently reported flexible electronic systems can infer the sensing parameter from the collected raw data, the majority of further data analysis is done by an external smart device. By leveraging on‐site data processing schemes such as TinyML, flexible electronic systems can benefit from local cognition, reduced latency and high power efficiency.

### 2.3 Power Management

To guarantee stable operation and long runtime, it is important to equip flexible electronic systems with sufficient power supply and management systems. Many have reported using a battery with a voltage regulator to provide a stable supply voltage. Lithium polymer battery is the most popular type of battery owing to its lightweight, high specific energy, and low form factor compared to other battery types such as lithium‐ion batteries.^[^ ^40^, ^175^, ^192^, ^203^, ^205^, ^251^, ^311^, ^312^, ^313^, ^314^ ^]^ There are also cases where lithium‐ion batteries^[^ ^9^, ^261^, ^315^ ^]^ or coin cells^[^ ^38^, ^39^, ^137^, ^157^, ^235^ ^]^ were used. The main advantage of using batteries as the power supply is its stability. In addition, with proper recharging medium and circuitry, it is possible to charge the battery.^[^ ^9^, ^205^, ^311^, ^312^, ^313^ ^]^ Regardless of the battery type, a voltage regulator module is needed to ensure a stable supply voltage to power the sensors, readout circuits, MCU, and BLE module. Typically, a low‐dropout voltage regulator (LDO) is used due to several benefits. First, because an LDO has a small voltage drop across the input and output, the majority of the battery's supply voltage can be utilized by the rest of the system. Second, compared to the output current, an LDO's quiescent current, which is the current consumed by the regulator, is almost negligible; this minimizes the regulator's power consumption. Furthermore, LDOs typically require fewer passive components in comparison to switch mode voltage regulators, which is beneficial from the miniaturization standpoint. If the battery's voltage is lower than the voltage required by an active component in the system, a boost converter should be used to meet the supply voltage requirement.^[^ ^137^, ^157^ ^]^

In place of batteries, energy harvesters can be utilized to power such systems. A popular choice is NFC‐based resonant magnetic inductive power transfer. An external NFC RF module generates an electromagnetic field at 13.56 MHz, which the receiving coil can absorb power from. Because the transferred power is an AC voltage, a rectifying circuit is needed to convert it into a DC voltage. Similar to the case where a battery powers the system, a suitable voltage regulator must be added at the output of the rectifier to ensure a stable supply voltage. Such a configuration is useful when the system has access to a constant RF field that can sufficiently power it or the whole system is to be implanted in biological tissue.^[^ ^204^, ^297^ ^]^ However, this configuration is dependent on the power transfer from an external NFC RF module; thus, ensuring a stable and constant coupling between the NFC RF module and the receiving coil on the system is critical. There are other energy harvesting methods such as photovoltaics,^[^ ^316^, ^317^, ^318^, ^319^, ^320^, ^321^ ^]^ thermoelectric generators,^[^ ^322^, ^323^ ^]^ triboelectric generators,^[^ ^98^, ^324^, ^325^, ^326^ ^]^ and piezoelectric generators.^[^ ^327^, ^328^, ^329^ ^]^ Sueng et al. demonstrated a peak voltage and current of 120 V and 65 µA, respectively, using a wearable triboelectric nanogenerator based on Ag‐coated textiles and ZnO nanopattern (Figure 3h).^[^ ^260^ ^]^ Yet, such energy harvesters still suffer from power supply instability arising in dynamic environments. It is possible to add a supercapacitor at the output of the voltage regulator to use it as a backup power source in the event of compromised energy harvesting (Figure 3i).^[^ ^55^, ^204^ ^]^ Nonetheless, if energy harvesting is insufficient to reliably power the system, then it may be prudent to use a battery as the main power supply and an energy harvester for charging the battery.

### 2.4 Actuation

As well as sensors, which are used to acquire data input, flexible electronic systems can feature actuators as the output for various purposes. Some sensors rely on the physiological response of the body when there is an external stimulus.^[^ ^10^, ^40^, ^41^, ^55^, ^315^, ^330^ ^]^ An example of this is skin hydration level estimation using the transient plane source (TPS) technique.^[^ ^40^, ^41^ ^]^ In this technique, heating elements and temperature sensors are placed closely. Voltage is applied across the skin‐interfaced heating elements, to induce heat fluxes to the skin and the nearby temperature sensors. Skin's thermal conductivity, which strongly depends on its hydration level, modulates how much heat flux goes into the skin, which can be inferred from the temporal change in the temperature sensors’ reading. A reference temperature sensor can be placed far from the thermal actuators to offset the influence of the ambient temperature.^[^ ^331^ ^]^ In addition, iontophoresis is important in sweat sensing because it can induce sweat on demand.^[^ ^332^, ^333^, ^334^ ^]^ Hydrogel‐containing agonist agents are applied at the iontophoresis electrodes. When electrical current is supplied to these electrodes, the agonist agents are transdermally transported to the sweat glands in the dermal layer, inducing sweat. Iontophoresis modules typically take several minutes to collect a sufficient amount of sweat. In addition, actuators are extensively used for therapeutics and pain relief using thermal, electrical, and optical stimulations.^[^ ^199^, ^207^, ^261^, ^297^, ^335^, ^336^, ^337^ ^]^ For instance, Song et al. reported using an anode electrode to induce an electric field on an open wound to promote keratinocyte migration to the wound site and thus accelerate the healing process.^[^ ^297^ ^]^ Zhang et al. showed the use of a PDMS needle array as the light guide to deliver UVA light into deep skin (Figure 3j).^[^ ^261^ ^]^ Furthermore, there were studies where LEDs were used for optogenetic neuromodulation of small animals^[^ ^204^, ^338^ ^]^ and biological cells.^[^ ^339^ ^]^

## 3 Integration Strategies

In Section 2, essential building blocks of standalone flexible electronic systems are delineated. In this section, different integration strategies to realize such systems are discussed. There are two extensively investigated approaches: flexible hybrid electronics and organic electronics. Flexible hybrid electronic systems feature flexible polymeric substrates, on which rigid surface‐mount devices are soldered. Organic electronic systems are constructed using flexible organic semiconducting materials for their channels.

Furthermore, tremendous effort has been exerted to realize all‐organic integrated circuits (ICs) through performance optimization and miniaturization of organic transistors. For the fabrication of organic transistors, various organic semiconductors haves been explored including pentacene, DNTT, PBTI, and P4.^[^ ^340^, ^341^, ^342^, ^343^ ^]^ However, several areas of enhancement still exist. First, organic semiconductors have inherently low electron and hole mobilities, which are reported to be up to 5.2 and 25 cm^2^V^−1^s^−1^, respectively, which are significantly lower than those of a monocrystalline Si.^[^ ^344^, ^345^ ^]^ Low carrier mobility limits the applicability of organic transistors by reducing their ON‐current. This necessitates the use of higher drive voltage in the range of tens of volts.^[^ ^19^ ^]^ Furthermore, many reported organic transistors have large dimensions, with channel lengths of tens of µm and channel widths of up to 1 mm.^[^ ^19^, ^344^, ^345^, ^346^, ^347^, ^348^ ^]^ This limits the miniaturization potential and thus reduces the integration density of all‐organic integrated circuits, resulting in a large system footprint. Therefore, from a performance and integration standpoint, the use of Si technology is preferred.

In addition, flexible transistors based on 2D materials are also gaining popularity in the community due to their high carrier mobility given the atomically thin thickness.^[^ ^349^ ^]^ However, the effective carrier mobility tends to be a lot lower than that of the theoretical limit. A benchmark study by Das et al. compares recently reported 10‐nm node Si FinFET, 10‐nm gate‐length CNT‐FET, and 29‐nm gate‐length MoS_2_ FET.^[^ ^350^ ^]^ In the study, all devices were calibrated such that their OFF‐current was equal to 10 nA FET^−1^, and then 17‐stage ring oscillator circuits were implemented to assess their delay and energy consumption. At all supply voltages, the Si FinFET exhibits the smallest delay per stage, energy per cycle, and leakage power, underlining the performance benefits and power efficiency of Si technology over the other two studied technologies. Graphene, as opposed to other 2D materials, has significantly higher carrier mobilities. However, uniformly depositing a monolayer of graphene over a large surface for large‐scale integration still remains a challenge. On the other hand, the fabrication technology for Si is highly mature, enabling cost‐effective and high‐integration‐density electronic components that are widely available. Therefore, in this review, the discussion of organic and novel‐material‐based electronics is omitted.

### 3.1 Flexible Hybrid Electronics

As briefly introduced, flexible hybrid electronics (FHE) refer to systems with a flexible polymeric substrate and rigid SMDs. In traditional electronics, a PCB, typically made of flame retardant 4 (FR4) and copper (Cu) interconnects,^[^ ^351^ ^]^ Similarly, in FHE, the rigid PCB is replaced with a flexible PCB (fPCB) consisting of polyimide (PI) and Cu interconnects. Commercially available PI films come in different thicknesses from 7.5 to 125 µm,^[^ ^352^ ^]^ while it is also possible to control the thickness by controlling the spin speed when spin‐coating PI precursors.^[^ ^353^ ^]^ In general, a lift‐off technique with photolithography and electron‐beam evaporation is used to pattern the Cu interconnects and bonding pads. Alternatively, PI films laminated with Cu layers are also commercially available.^[^ ^354^ ^]^ To ensure the reliability of Cu interconnects during bending cycles, it is a common practice to use serpentine or horseshoe designs. In addition to Cu interconnects and bonding pads, standard microfabrication techniques, and laser ablation can be used to directly fabricate sensors based on thin‐film metals and LIG, respectively, on the PI substrate. It should be noted that commercial COTS SMD sensors are also widely used to facilitate the integration process.^[^ ^11^, ^12^, ^37^, ^38^, ^40^, ^41^, ^205^, ^206^, ^244^, ^252^, ^313^, ^314^, ^355^, ^356^, ^357^, ^358^, ^359^ ^]^ Then, SMDs can be soldered on the fPCB to build the electronics assembly with front‐end circuitry, MCU, wireless communication module and power management circuitry. Finally, soft silicone‐based elastomers such as PDMS or EcoFlex are used to encapsulate the system.

Healthcare devices made with FHE can be categorized into two groups: implantable and wearable. In implantable devices, the sensor node or the entire system is implanted inside the body. For some applications such as pacemakers^[^ ^199^, ^360^ ^]^ and organ transplant rejection monitoring,^[^ ^39^ ^]^ implantable devices are a necessity, but implantable devices can be also used for monitoring physiological parameters that can be alternatively measured using wearable technology for enhanced sensing accuracy and robustness. However, implantable devices require implantable procedures, which may introduce infection risks, and can often cause discomfort. Therefore, implantable devices are often incorporated during surgery.

**Figure 4:**
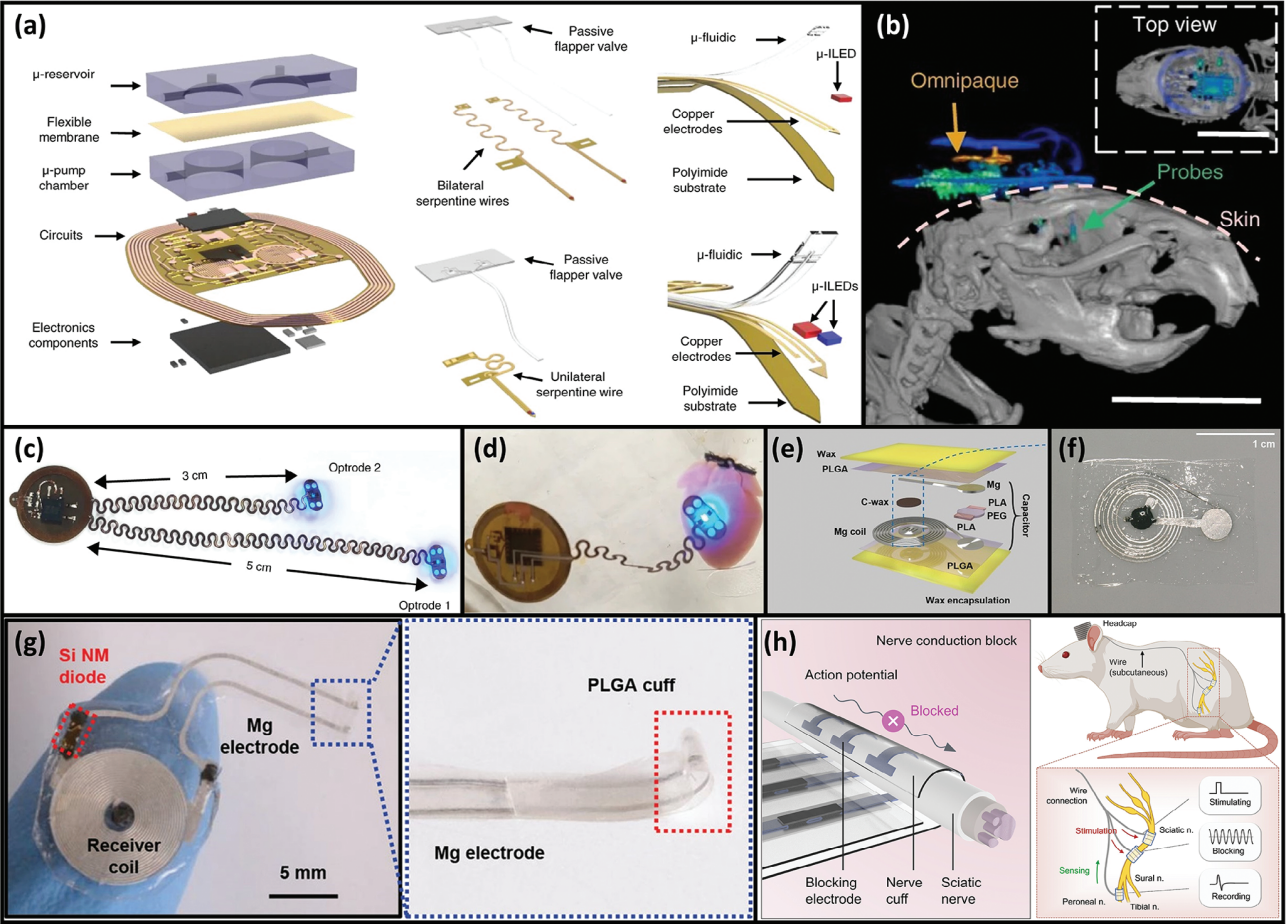
Implantable flexible hybrid electronics. a) Exploded view of a semi‐implantable optogenetic and pharmacological neuromodulation device. b) CT image of the mouse with the device implanted. a,b) Reproduced under the terms of the CC‐BY Creative Commons Attribution 4.0 International license.^[^ ^338^ ^]^ Copyright 2022, The Authors, published by Springer Nature. c) Optical image of an implantable optogenetic pacemaker. d) Optical image of the device in use. c,d) Reproduced under the terms of the CC‐BY Creative Commons Attribution 4.0 International license.^[^ ^199^ ^]^ Copyright 2019, The Authors, published by Springer Nature. e) Exploded view of a transient implantable internal body temperature sensor. f) Optical image of the device before the final encapsulation with wax. e,f) Reproduced with permission.^[^ ^361^ ^]^ Copyright 2020, Wiley‐VCH Verlag GmbH & Co. KGaA, Weinheim. g) Optical image of an implantable electrical stimulator for peripheral axon regeneration. Reproduced with permission.^[^ ^362^ ^]^ Copyright 2021, Wiley‐VCH. h) Illustration of the structure of an implantable electronic peripheral nerve pain blocker. Reproduced under the terms of the CC‐BY Creative Commons Attribution 4.0 International license.^[^ ^336^ ^]^ Copyright 2022, The Authors, some rights reserved; exclusive licensee AAAS, published by AAAS.

Implantable devices can take different forms depending on the degree to which they are implanted into the human or animal body. Semi‐implantable devices refer to devices whose sensor or actuator node is inserted into the body while the electronics module is epidermally mounted or exposed to the outside.^[^ ^315^, ^338^, ^363^ ^]^ An example of such a device is the semi‐implantable device reported by Guo et al. for interstitial oxygen saturation of tissues.^[^ ^315^ ^]^ The device consists of a skin‐mounted electronics module with a flexible optically actuating/sensing probe with SMD µ‐LEDs and µ‐PDs for PPG measurements, from which the local tissue oxygenation (StO_2_) can be derived. Also, Wu et al. developed a miniaturized implantable device for small animals featuring optogenetic and microfluidics‐enabled pharmacological neuromodulating capabilities (see **Figure** **4**a,b).^[^ ^338^ ^]^

Unlike semi‐implantable devices, conventional implantable devices are inserted into the body as a whole, which necessitates the removal of the device after the operational period. Gutruf et al. fabricated a wirelessly powered implantable optogenetic pacemaker for small animals.^[^ ^199^ ^]^ The device features two platinum (Pt) electrodes, which are fabricated on a PI substrate and connected to the electronics assembly through serpentine interconnects as shown in Figure 4c. The Pt electrodes monitor ECG signals while the system performs optogenetic pacemaking via the µ‐LEDs mounted next to the Pt electrodes (Figure 4d). However, as mentioned above, traditional implantable devices as such involve highly invasive post‐operation surgery for removal, increasing infection risks and prolonging the recovery process of surgical wounds.

To circumvent the need for a second surgery to remove the implanted device, many transient sensors and actuators based on bioresorbable materials such as poly(lactic‐co‐glycolic acid) (PLGA) and molybdenum (Mo) have been reported.^[^ ^203^, ^297^, ^336^ ^]^ Some examples of bioresorbable devices include internal body temperature sensors (Figure 4e,f) and electrical nerve stimulators (Figure 4g,h).^[^ ^361^ ^]^

Because transient devices are designed to be harmlessly absorbed into the body, it is difficult to incorporate conventional non‐bioresorbable materials or electronic components in such devices and make them standalone. Thus, they need to be assisted with a complementary module that can supply power and perform data management. This is typically done by epidermally mounting a wearable module—capable of wirelessly supplying power and communicating—near the implanted device's location. A dual‐module system as such can be categorized as implantable/wearable hybrids. Choi et al. reported such a system, where the chest‐mounted cardiac module powers and controls the implanted module for electrical pacing via NFC.^[^ ^203^ ^]^

Wearable solutions are gently mounted on the skin of the user to measure various physiological markers and are thus non‐invasive by nature. Unlike implantable devices, the application and removal of a wearable device are facile, making it versatile and advantageous for at‐home or daily applications.^[^ ^10^, ^175^, ^296^ ^]^

**Figure 5:**
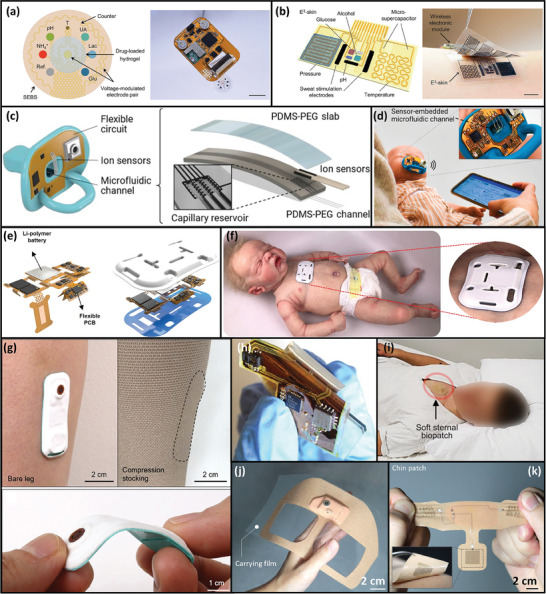
Wearable flexible hybrid electronics. a) Left: Illustration of the sweat‐sensing node of a wearable multiplexed biosensing platform. Right: Optical image of the entire system consisting of a flexible hybrid electronics module and the sensor node. Reproduced under the terms of the CC‐BY Creative Commons Attribution 4.0 International license.^[^ ^235^ ^]^ Copyright 2023, The Authors, some rights reserved; exclusive licensee AAAS, published by AAAS. b) Left: 3D model of the 3D‐printed sensor/supercapacitor node of an integrated physiological monitoring device. Right: Optical image of the device. Reproduced under the terms of the CC‐BY Creative Commons Attribution 4.0 International license.^[^ ^55^ ^]^ Copyright 2023, The Authors, some rights reserved; exclusive licensee AAAS, published by AAAS. c) Left: 3D model of a pacifier functionalized with a microfluidics‐enabled saliva electrolyte sensor. Right: Exploded view of the microfluidics sensing node. d) Optical image of the pacifier in use. Inset: Close‐up image of the device's electronics module. c,d) Reproduced with permission.^[^ ^237^ ^]^ Copyright 2022, Elsevier B.V. e) Exploded view of a physiological monitoring platform for neonates. f) Optical image of the device mounted on a neonate's chest. Inset: Close‐up image of the device. e,f) Reproduced with permission.^[^ ^205^ ^]^ Copyright 2021, Wiley‐VCH GmbH. g) Top: Optical of the device in use. Bottom: Optical image of the device under a bending test at a being radius of 30 mm. Reproduced under the terms of the CC‐BY Creative Commons Attribution 4.0 International license.^[^ ^38^ ^]^ Copyright 2021, Wiley‐VCH GmbH. h) Optical image of a chest‐mounted device for sleep apnea detection and sleep stage classification under a bending test. i) Optical image of the device in use. h,i) Reproduced under the terms of the CC‐BY Creative Commons Attribution 4.0 International license.^[^ ^296^ ^]^ Copyright 2021, The Authors, some rights reserved; exclusive licensee AAAS, published by AAAS. j) Optical image of the forehead module of a device for sleep quality and sleep apnea assessment. k) Optical image of the chin module of the device. j,k) Reproduced under the terms of the CC‐BY Creative Commons Attribution 4.0 International license.^[^ ^175^ ^]^ Copyright 2023, The Authors, some rights reserved; exclusive licensee AAAS, published by AAAS.

Chemical physiological sensing of biofluids such as interstitial fluids, sweat, and saliva can be efficiently done with wearable sensing platforms. Shirzaei Sani et al. fabricated an interstitial fluid sensing patch for chronic wound monitoring and therapy as shown in **Figure** **5**a.^[^ ^235^ ^]^ The device measures NH_4_^+^, pH, uric acid, lactate, and glucose levels of the interstitial fluid and the temperature of the wound site. It can also accelerate the wound healing process by electrical stimulation and iontophoresis‐based antimicrobial drug delivery.

Another branch of chemical sensing extensively investigated is sweat sensing. Song et al. additively fabricated a multimodal sweat sensing platform powered by a flexible solar cell with supercapacitors as temporary energy storage (see Figure 5b).^[^ ^55^ ^]^ The device can measure the pH, glucose and alcohol levels of sweat induced by iontophoresis with carbachol hydrogel as well as pressure and temperature. As discussed in Section 2.1.7, the aforementioned chemical sensors are integrated into a single microfluidic reservoir, which increases the sensor density, by having multiple electrodes with different interface materials. The reference electrode (RE) is made of Ag, while the working electrodes (WEs) are decorated with materials sensitive to each specific ion or compound. Utilizing similar principles, Lim et al. functionalized a commercially available pacifier with a microfluidics/electronics module for neonate's saliva electrolyte analysis such as Na^+^ and K^+^ (see Figure 5c,d).^[^ ^237^ ^]^

Additional markers apart from biofluid ions and compounds are also useful in physiological monitoring: temperature, pressure, biopotentials, etc. Figure 5e shows another neonatal wearable multimodal monitoring device capable of measuring ECG, temperature, respiratory, and heart rates.^[^ ^205^ ^]^ The device relies on SMD sensors such as a digital temperature sensor and a triaxial accelerometer. Park et al. reported a system featuring an SMD temperature sensor and a 3D‐buckled Au‐based piezoresistive pressure sensor for continuous monitoring of temperature and pressure between the skin and therapeutic compression garment (see Figure 5g).^[^ ^38^ ^]^ While using off‐the‐shelf SMD components can facilitate the system integration process and provide reliable measurements, the rigidity of such components—along with those for front‐end circuits, data, and power management—can compromise the overall flexibility, necessitating judicious design techniques (see Figure 5f–h).

In addition to daytime monitoring, wearable sensors can be used to track physiological status during sleep. Kwon et al. reported an epidermal sleep monitoring system estimating sleep quality and predicting sleep apnea (see Figure 5j–k).^[^ ^175^ ^]^ Sleep apnea is predicted from the measured EEG, EOG, and EMG measured from different sites on a face. Because the prediction of sleep apnea and quality requires frequency domain analysis and convolutional neural network, both of which demand heavy computational load, as mentioned in Section 2.2.2, the computation is done by a nearby smart device receiving the raw data from the monitoring system via BLE.

In addition to the hitherto discussed well‐known sensing methods, it is possible to map internal deep tissue using the acoustic resonance technique. Lin et al. reported a skin‐mounted ultrasound patch monitoring central blood pressure, heart rate, and cardiac output via an array of flexible piezoelectric transducers fabricated by microfabrication techniques, laser ablation, and transfer printing.^[^ ^364^ ^]^ The transducer array has a different center frequency of 2–6 MHz depending on its design, yielding different axial resolutions (600, 300, and 230 µm, respectively) and penetration depths (164, 78, and 9 mm, respectively). Different array modules can be connected to the electronics assembly to image different tissues located in different parts and depths of a human body (e.g., diaphragm, heart, carotid artery).

Other reported FHE devices are summarized in **Table** **4**. As seen in the examples above, in FHE, thin flexible sensor nodes can conformally adhere to most curvilinear surfaces such as human skin, providing good contact and thus ensuring reliable collection of data. Furthermore, due to the use of mature CMOS technology, FHE systems exhibit good performance: high‐precision conversion of sensed analog data, fast data processing and facile actuation, and wireless communication with external devices. However, the rigid SMDs used in the electronic assembly pose a bottleneck in terms of flexibility (i.e., bending radius) simply because rigid components cannot be bent. Furthermore, the packaging material encapsulating each SMD increases the overall footprint of the system, which is disadvantageous from a miniaturization standpoint.

**Table 4:** Summary of reported flexible hybrid electronics.

Refs.	Application	Type	Sensors	Communication	Power	Actuation	Minimum Bending Radius [mm]	Dimensions (W × L × H) [mm]
[365]	Scalp microneedle for human‐machine	Semi‐implantable	EEG (potentiometric, Au microneedle electrode array, AFE)	BLE	Battery	–	1.5 (interconnect only) 5 (Au microneedle electrode array only)	Each Au microneedle electrode array: 6 × 6 × 1.15 Electronics module: –
[315]	Local tissue oxygenation monitoring	Semi‐implantable	StO_2_ (optical, SMD PD, SMD LED, SMD TIA)	BLE	Lithium‐ion battery	–	–	11.1 × 20 × ‐
[338]	Neuromodulation of small animals	Semi‐implantable	–	NFC	Electromagnetic induction	Optogenetic neuromodulation (SMD µ‐LED) Pharmacological neuromodulation (microfluidic drug delivery)	–	10 × 13 × 5.5
[261]	Deep‐skin UVA therapy	Semi‐implantable	–	–	External wired power supply	UVA emission (SMD UVA LEDs, PLGA microneedle waveguide)	3.5	10 × 10 × 3.4
[363]	Microneedle patch for human‐machine interface	Semi‐implantable	EMG (potentiometric, Au microneedle array)	BLE	Lithium‐polymer battery	–	–	–
[204]	Neuromodulation of small animals	Implantable	EEG (potentiometric, Au electrodes, SMD AFE) EMG (potentiometric, stainless steel electrodes, SMD AFE)	NFC	Electromagnetic induction with supercapacitors	Optogenetic neuromodulation (SMD µ‐LED) Pharmacological neuromodulation (microfluidic drug delivery)	–	40 × 18 × 3
[299]	Vascular monitoring	Implantable	Blood flow (piezoresistive, Si NM) Pressure (piezoresistive, Si NM) Temperature (thermoresistive, Si NM)	BLE	Electromagnetic induction with supercapacitors	–	–	Sensor node: 3 × 8 × 2.3 Electronics module: 33 × 33 × 3
[330]	Local tissue oxygenation monitoring	Implantable	StO_2_ (optical, SMD PD, SMD LED, SMD TIA)	IR communication	Electromagnetic induction with supercapacitors	–	–	13 × 10 × 1
[366]	Neuromodulation of small animals	Implantable	–	NFC	Electromagnetic induction	Optogenetic neuromodulation (SMD µ‐LED)	–	8 × 8 × 1
[203]	Physiological monitoring and cardiac pacing	Implantable/ Wearable	Cardiac module: ECG (potentiometric, Cu electrode, SMD AFE) RR and HR (acceleration, SMD IMU) Respiration module: RR and HR (acceleration, SMD IMU) Hemodynamic module: SpO_2_ (optical, SMD PD, SMD LED, SMD TIA)	BLE	Wearable modules: Lithium‐polymer battery Implantable module: Electromagnetic induction	Implantable module: Cardiac pacing (Mo electrode) Haptic module: Haptic feedback (eccentric rotating mass actuators)	2.2 (interconnect only)	–
[355]	Cardiovascular monitoring	Wearable	Pressure (piezoresistive, flexible 3D buckled sensor) Temperature (thermoresistive, SMD thermistor)	BLE	Coin cell battery	–	–	50 × 25 × 3.5
[37]	Wound monitoring	Wearable	Temperature (thermoresistive, SMD thermistor)	BLE or NFC	Battery or Electromagnetic induction	Heating for thermal measurement (SMD resistive heating element)	50	–
[40]	Skin hydration sensor	Wearable	Temperature (thermoresistive, SMD thermistor)	BLE	Lithium‐polymer battery	Heating for thermal measurement (SMD resistive heating element)	4 (interconnect only)	30 × 15 × 6
[297]	Wound monitoring	Wearable	Wound healing progress (resistive, bioresorbable Mo electrodes)	NFC	Electromagnetic induction	Electrical stimulation (Mo electrodes)	–	–
[311]	Mechano‐acoustic monitoring	Wearable	Body movement (acceleration, SMD IMU) One of the following depending on the sensor location: Intestinal, cardiac and respiratory sounds (SMD microphones)	BLE	Lithium‐polymer battery	–	–	40 × 20 × 8
[205]	Physiological monitoring for neonatal patients	Wearable	ECG (potentiometric, Au electrodes, SMD AFE) Temperature (thermoresistive, SMD digital temperature sensor) RR and HR (acceleration, accelerometer)	BLE	Lithium‐polymer battery	–	22	–
[206]	Physiological monitoring for pediatric patients	Wearable	Chest module: ECG (potentiometric, Au electrodes, SMD AFE) RR, SCG, body orientation, activity (acceleration, accelerometer) Limb module: SpO_2_ (optical, SMD PD, SMD LED, SMD TIA) Temperature (thermoresistive, SMD digital temperature sensor)	BLE	Lithium‐polymer battery	–	5.5 (interconnect only)	Chest module: 50 × 30 × 6 Limb module: 90 × 30 × 6
[313]	Cardiopulmonary monitoring	Wearable	RR, HR, temperature, activity (differential acceleration, 2 IMUs)	BLE	Lithium‐polymer battery	–	–	46 × 22 × 9
[38]	Physiological monitoring for compression therapy patients	Wearable	Pressure (piezoresistive, flexible 3D buckled sensor) Temperature (thermoresistive, SMD thermistor)	BLE	Coin cell battery	–	30	20 × 35 × 2.5
[41]	Skin hydration sensor	Wearable	Temperature (thermoresistive, SMD thermistor)	NFC	Electromagnetic induction	Heating for thermal measurement (SMD resistive heating element)	10	45 × 31 × 2.1
[252]	Physiological monitoring for neonatal and pediatric patients	Wearable	Chest module: ECG (potentiometric, Au electrodes, SMD AFE) RR, HR, SCG, activity (acceleration, accelerometer) Temperature (thermoresistive, SMD digital temperature sensor) Limb module: SpO_2_ (optical, SMD PD, SMD LED, SMD TIA) Temperature (thermoresistive, SMD digital temperature sensor)	BLE	Coin cell battery or lithium‐polymer battery or electromagnetic induction	–	20	56 × 22 × ‐
[9]	Mechano‐acoustic monitoring	Wearable	RR, HR, activity (acceleration, accelerometer)	BLE	Lithium‐ion battery Wireless charging	–	–	36 × 21 × ‐
[367]	Neuromuscular function monitoring	Wearable	EMG (potentiometric, Ag/AgCl electrodes, SMD AFE) Activity (acceleration, accelerometer)	BLE	Lithium‐ion battery Wireless charging	–	–	34 × 66 × 3.5
[368]	Cardiac monitoring	Wearable	ECG (potentiometric, Au electrodes, SMD AFE)	NFC	Custom‐designed battery Electromagnetic induction	–	100	55 × 25 × 1
[369]	Ventricular shunt function monitoring	Wearable	Temperature (thermoresistive, flexible Au sensor array)	BLE	Lithium‐polymer battery	Heating for thermal measurement (flexible Au resistive heating element)	–	18 × 41 × 4
[356]	Sensor node for spatial mapping of pressure and temperature	Wearable	Temperature (thermoresistive, SMD NFC chip) Pressure (piezoresistive, Si NM)	NFC	Electromagnetic induction	–	–	14.6 × 14.6 × 0.6
[10]	Stress monitoring based on sweat sensing	Wearable	Sweat Na^+^, K^+^, NH_4_^+^ (amperometric, C electrodes, SMD INA) GSR (resistive, Ag electrodes) Temperature (thermoresistive, C electrode) Sweat glucose, lactate, uric acid (amperometric, C electrodes with enzymatic coating) Pulse (piezocapacitive, Ag electrodes, PDMS‐engraved airgap)	BLE	Battery	IP for local sweat induction (C electrodes, carbachol hydrogel at the anode)	–	38 × 30 × 2
[137]	Monitoring of C‐reactive protein in sweat	Wearable	Temperature (thermoresistive, LIG electrode) Sweat pH (potentiometric, LIG electrode coated with PANI) C‐reactive protein (potentiometric, LIG electrode) Sweat ionic strength (impedimetric, LIG electrodes)	BLE	Coin cell battery	IP for local sweat induction (LIG electrodes, carbachol hydrogel at the anode)	25	31.7 × 25.5 × 3
[157]	Ring device for female hormone monitoring	Wearable	Temperature (thermoresistive, SMD AFE) Sweat pH (potentiometric, PANI electrodes) Sweat ionic strength (Impedimetric, MXene‐Au NP electrode)	BLE	Coin cell battery	IP for local sweat induction (C and Ag electrodes, carbachol hydrogel at the anode)	10	13 × 67 × 2
[55]	3D‐printed sweat sensing for health monitoring	Wearable	Pressure (piezoresistive, CNT‐PDMS and MXene electrodes) Sweat glucose (CNT‐SBS electrodes with chitosan and glucose oxidase) Sweat alcohol (CNT‐SBS electrodes with chitosan and alcohol oxidase) Temperature (thermoresistive, MXene electrode) Sweat pH (potentiometric, PANI electrode)	BLE	Flexible solar cell with MXene supercapacitors	IP for local sweat induction (CNT‐SBS electrodes, gelatin‐agarose‐carbachol hydrogel at the anode)	20	–
[235]	Chronic wound monitoring	Wearable	Glucose, lactate, uric acid (amperometric, Au electrodes with enzymatic coating) pH (potentiometric, Au electrode with PANI) Temperature (thermoresistive, Au electrode) Ammonium (potentiometric, Au electrode with ammonium ionophore I)	BLE	2 coin cell batteries	IP for drug delivery (Au electrodes, hydrogel loaded with antimicrobial drug at the node) Electrical stimulation (same Au electrodes as the IP drug delivery)	10	36.5 × 25.5 × 2
[56]	Physiological monitoring	Wearable	Temperature (thermoresistive, LIG electrode) Sweat amino acids (amperometric, LIG electrodes with molecularly imprinted polymer and additive enzymes)	BLE	Battery	IP for local sweat induction (LIG electrodes, carbachol hydrogel at the anode)	–	21 × 49 × 2
[357]	Physiological monitoring/human‐machine interface	Wearable	Urea, NH_4_^+^, glucose (potentiometric, Au‐based ion selective electrodes) pH (potentiometric, Au electrode with PANI) Temperature (thermoresistive, SMD BLE module)	BLE	Lactate biofuel cell (power density = 3.5 mWcm^−2^)	–	15	30 × 39 × 3
[244]	Physiological monitoring	Wearable	Tyrosine, uric acid (amperometric, LIG electrodes with enzymatic coating) Strain (piezoresistive, porous LIG electrode) Temperature (thermoresistive, LIG electrode)	BLE	Lithium‐ion battery	–	–	24 × 60 × 3
[158]	Physiological monitoring	Wearable	pH (potentiometric, Au electrode with PANI coating) Na^+^ (potentiometric, Au‐based ion‐selective electrode)	BLE	Flexible TENG (power density = 0.0416 mW	–	20	Sensor/Electronics module: 26 × 35 × 2 Flexible TENG module: 63 × 120 × 0.24
[370]	Hand gesture recognition for human‐machine interface	Wearable	EMG (potentiometric, Au electrode array)	BLE	Lithium‐polymer battery	–	45	–
[11]	Physiological monitoring for postpartum women	Wearable	SpO_2_ (optical, SMD PPG sensor) ECG (potentiometric, Au electrodes, AFE) Temperature (thermoresistive, SMD temperature sensor)	BLE	Battery	–	–	–
[175]	Sleep monitoring	Wearable	Forehead module: EEG (potentiometric, Au electrodes, AFE) EOG (potentiometric, Au electrodes, AFE) Chin module: EMG (potentiometric, Au electrodes, AFE)	BLE	Lithium‐polymer battery	–	–	Forehead module: 32 × 19 × – (electronics module) 96 × 70 × ‐ (entire module) Chin module: 32 × 19 × – (electronics module) 56 × 39 × ‐
[251]	Fall monitoring for elderly people	Wearable	Movement (acceleration, IMU)	BLE	Lithium‐polymer battery	–	2	41 × 28 × ‐
[305]	Stress monitoring based on cardiac measurement	Wearable	SCG (acceleration, accelerometer)	BLE	Lithium‐polymer battery	–	1.5 (interconnect only)	–
[314]	Stress monitoring and radiative cooling for outdoor applications	Wearable	GSR (resistive, Au electrodes) Temperature (thermoresistive, SMD thermistor)	BLE	Lithium‐polymer battery Wired charging	Passive cooling of electronics module (nanofabric radiative cooler)	–	85 × 80 × 5.3
[371]	Physiological monitoring during exosuit usage	Wearable	ECG (potentiometric, Au electrodes) Movement (acceleration, IMU)	BLE	Lithium‐polymer battery	–	–	25 × 14 × ‐
[237]	Saliva electrolyte monitoring for neonates	Wearable	Na^+^, K^+^ (potentiometric, CB/Ecoflex composite electrodes with ion‐selective materials)	BLE	Rechargeable battery	–	–	–
[372]	Portable digital stethoscope	Wearable	Cardiac and respiratory sounds (piezocapacitive, SMD MEMS microphone)	BLE	Lithium‐polymer battery Wired charging	–	–	–
[358]	Physiological monitoring for outdoor workers	Wearable	ECG, HR (potentiometric, Au electrodes, AFE) Temperature (thermoresistive, SMD digital temperature sensor) Activity (acceleration, SMD IMU)	BLE	Lithium‐polymer battery Wired charging	–	10.4	90 × 3.5 × 5
[373]	Cardiac monitoring with improved noise suppression from sweat and temperature	Wearable	ECG (potentiometric, Au electrodes, AFE)	BLE	Lithium‐polymer battery Wired charging	–	35	100 × 45 × 7.1
[35]	Physiological monitoring with EMG interference suppression	Wearable	ECG, HR, RR (potentiometric, Au electrodes, AFE) Activity (acceleration, accelerometer)	BLE	Lithium‐ion battery	–	15	44 × 8 × ‐
[296]	Sleep monitoring	Wearable	ECG (potentiometric, Au electrodes, AFE) SCG, Activity (acceleration, accelerometer) SpO_2_ (optical, SMD PD, SMD LED)	BLE	Lithium‐polymer battery	–	3 (interconnect only)	22 × 43 × ‐
[167]	Neuromuscular monitoring for human‐machine interface	Wearable	EMG (potentiometric, graphene electrodes)	BLE	Lithium‐polymer battery	–	1.5 (interconnect only)	35 × 65 × 2
[190]	Eye vergence tracking for virtual reality	Wearable	EOG (potentiometric, Au electrodes)	BLE	Lithium‐polymer battery	–	15	36 × 54 × 8
[359]	Physiological monitoring	Wearable	GSR (resistive, Au electrodes) Temperature (thermoresistive, SMD digital temperature sensor)	BLE	Lithium‐polymer battery	–	–	31 × 22 × 2
[253]	Physiological monitoring and abnormality detection	Wearable	ECG, HR, RR (potentiometric, Au electrodes, AFE) Activity (acceleration, accelerometer)	BLE	Lithium‐polymer battery	–	12.5	Electronics module: 19.9 × 32.7 × 2 Entire system: 45 × 45 × 7
[374]	Physiological monitoring for cervical dystonia diagnosis	Wearable	Head motion (acceleration, accelerometer)	BLE	Lithium‐polymer battery	–	10	19.9 × 32.7 × ‐
[24]	Intraoral sodium intake monitoring	Wearable	Na^+^ (potentiometric, Cu/Pd electrode with Na^+^ membrane)	BLE	Coin cell battery	–	5	27 × 21 × 2.5
[375]	Ultrasound monitoring for deep tissue modulus mapping	Wearable	Deep tissue modulus mapping (acoustic resonance, Cu electrode array with piezoelectric transducers)	Wired communication	External wired power supply	–	–	–
[364]	Wearable ultrasound monitoring	Wearable	Deep tissue modulus mapping (acoustic resonance, Cu electrode array with piezoelectric transducers)	Wi‐Fi	Lithium‐polymer battery	–	60	21 × 41 × 2
[376]	Sweat sensor integrated with stretchable battery	Wearable	Na^+^, pH (potentiometric, C electrodes)	–	Stretchable Ag_2_O‐Zn battery	Electrochromic display (PEDOT:PSS)	–	20 × 20 × 1
[12]	3D stretchable electronics module	Wearable	Temperature (thermoresistive, SMD thermistor) Strain (piezoresistive, flexible strain sensor) Orientation (SMD gyroscope) Movement (SMD accelerometer)	BLE	Pouch cell	–	–	30 × 19 × 2
[4]	Tear exosome detection	Wearable	pH in 6.5‐7.4 range (chemical, Au NP colorimetric assay)	–	–	Colorimetric feedback on pH (Au NP)	–	–
[5]	Intraocular pressure monitoring	Wearable	Pressure (piezocapacitive, AgSEBS electrodes, Silbione)	RF resonance	External analyzer	Output read by external RF analyzer	–	–
[6]	Ocular electrodiagnosis	Wearable	ERG (potentiometric, AgSEBS‐Au‐PEDOT electrode)	Wired communication	External analyzer	Output read by external RF analyzer	–	–

Abbreviations: AFE, analog front‐end; BLE, Bluetooth Low Energy; C, carbon; CB, carbon black; ECG, electrocardiogram; EEG, electroencephalogram; EMG, electromyogram; EOG, electrooculogram; ERG, electroretinogram; GSR, Galvanic skin response; HR, heart rate; IMU, inertial measurement unit; INA, instrumentation amplifier; IP, iontophoresis; IR, infrared; LED, light‐emitting diode; LIG, Laser‐induced graphene; MXene, Ti3C2Tx; NFC, near‐field communication; NM, nanomembrane; NP, nanoparticle; PANI, polyaniline; PD, photodiode; PDMS, polydimethylsiloxane; PEDOT:PSS, poly(3,4‐ethylenedioxythiophene):poly(styrene sulfonate); PLGA, poly(lactic‐co‐glycolic acid); RR, respiratory rate; SBS, poly(styrene‐butadiene‐styrene); SCG, seismocardiogram; SEBS, poly(styrene‐ethylene‐butylene‐styrene); SMD, surface‐mount device; SpO2, blood oxygen saturation; StO2, tissue oxygen saturation; TIA, transimpedance amplifier; UVA, ultraviolet spectrum with wavelength range of 315–400 nm.

### 3.2 Transformational Electronics

As discussed so far, flexible electronic systems require two important aspects: flexibility and performance. Organic electronics exhibit excellent flexibility as well as stretchability, but their integration density and performance tend to be limited compared to those of state‐of‐the‐art CMOS technology. On the other hand, due to the use of state‐of‐the‐art CMOS components, FHE systems possess improved integration density, power efficiency, and performance. Yet, despite the use of low‐profile SMD packaging, the rigidity of individual electronic components imposes a fundamental limit on FHE systems’ flexibility. Furthermore, the lateral dimensions of SMD packaging increase the minimum required footprint of the whole system. Thus, in this section, a third integration strategy—referred to as transformational electronics (TE)—is discussed.

The central idea behind TE is similar to that of FHE—the use of state‐of‐the‐art CMOS technology. However, the differentiating factor lies within the integration scheme. While FHE systems consist of rigid packaged SMDs, TE systems contain bare‐die components, which possess a much smaller footprint compared to SMDs. For instance, ADS1298 is a widely used medical AFE component—typically in quad flat pack (QFP) packaging. The QFP version of ADS1298 has dimensions of 12 × 12 × 1.2 (L × W × H) mm^3^, whereas the bare‐die version has much smaller dimensions of 5.639 × 6.045 × 0.28 mm^3^.^[^ ^377^, ^378^ ^]^ Furthermore, by utilizing customized ASICs, it is possible to incorporate novel materials and device architecture for experimental systems. For example, hybrid memory devices based on molecular oxides such as polyoxometalates (POMs) can exhibit high data density and have the potential to be integrated in CMOS systems.^[^ ^379^ ^]^ However, it is yet to be adopted by the semiconductor industry due to process challenges at an industrial scale.^[^ ^380^ ^]^ The bare‐die integration of customized ASICs based on such novel devices alleviates the dependence on the available COTS components. Hence, this allows the assessment of the efficacy and feasibility of such novel devices at a system level, expanding the arsenal of transformational electronics.

Heterogenous integration of such small bare‐die components boosts the integration density and thus reduces the overall footprint of the final system, which is a critical factor in many applications of flexible electronics where real estate is limited. Khan et al. fabricated a flexible IoT device for plant monitoring.^[^ ^381^ ^]^ The system contains an ADC/DAC ASIC, MCU, and solid‐state battery—all in bare‐die forms—as well as a flexible humidity, temperature, and light sensor. The system has overall dimensions of 12 × 28 × 0.35 mm^3^.

**Figure 6:**
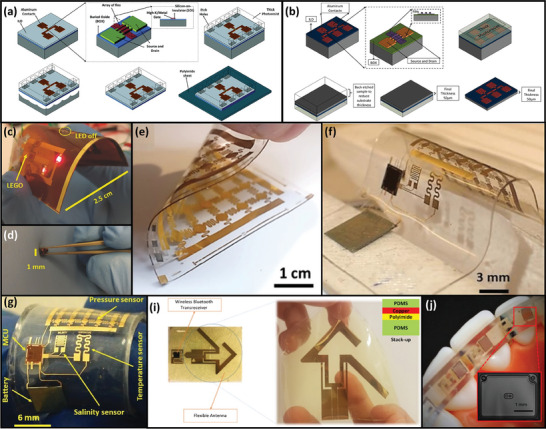
Transformational Electronics. a) Fabrication flow of xenon difluoride (XeF_2_)‐based Si fabric release (i.e., “trench‐protect‐release‐reuse” method). Reproduced with permission.^[^ ^382^ ^]^ Copyright 2014, Wiley‐VCH. b) Fabrication flow of deep reactive ion etching (DRIE)‐based Si thinning (i.e., “sequential back‐etch” method). Reproduced with permission.^[^ ^383^ ^]^ Copyright 2014, ACS. c) Optical image of LEGO‐assembled flexible system under a bending test. d) Optical image of an individual LEGO bare‐die module under a bending test at a bending radius of 0.5 mm. Reproduced with permission.^[^ ^29^ ^]^ Copyright 2017, Wiley‐VCH. e,f) Optical images of a bare‐die‐integrated lightweight marine tagging system. g) Optical image of the device with annotation for different parts. h) Optical image of the device under a bending test. Reproduced with permission.^[^ ^384^ ^]^ Copyright 2019, Wiley‐VCH. i) Left: Optical image of a bare‐die‐integrated BLE module with a flexible antenna. Right: Optical image of the flexible antenna. Reproduced under the terms of the CC‐BY Creative Commons Attribution 4.0 International license.^[^ ^385^ ^]^ Copyright 2019, IOP Publishing. j) Optical image of a miniaturized orthodontic system featuring solid‐state microbatteries with an ultra‐low profile. Reproduced under the terms of the CC‐BY Creative Commons Attribution 4.0 International license.^[^ ^386^ ^]^ Copyright 2017, The Authors, published by Springer Nature.

Furthermore, by using microfabrication techniques, it is possible to reduce a bare die's thickness, which can improve the flexibility of the overall system. There are two reported techniques to transform a rigid Si CMOS device into a flexible form. First is the “trench‐protect‐release‐reuse” method.^[^ ^387^ ^]^ This technique consists of the following steps. First, a device is fabricated on a rigid Si wafer. Trenches are formed in non‐functional areas of the wafer using DRIE (trench step). Then, hard mask materials resistant to fluorine such as silicon oxide (SiO_2_) or aluminum oxide (Al_2_O_3_) are deposited using atomic layer deposition (ALD). The hard mask deposited on the horizontal surface is removed by RIE (protect step). Finally, the top functional layer is released by isotropically etching Si using XeF_2_ (release step). The bottom Si wafer can be planarized by chemical mechanical polishing (CMP) and reused for subsequent device fabrication (reuse step).^[^ ^388^ ^]^ Many devices including metal‐oxide‐semiconductor capacitors (MOSCAPs),^[^ ^387^, ^389^ ^]^ random access memory (RAM),^[^ ^390^, ^391^ ^]^ FinFET,^[^ ^382^ ^]^ and microbattery (see **Figure** **6**a).^[^ ^392^ ^]^ The main advantage of this method is that any Si device can be flexed over a large area given there is sufficient non‐functional area for trench formation. Furthermore, the bottom Si wafer after the release step can be reused, improving its cost‐effectiveness. However, the main disadvantage of this approach is the area loss caused by trench formation.

Instead, an alternative denoted as “sequential back‐etch” can be used. Similar to the previous method, the functional layer is first fabricated before the flexing step. The substrate (e.g., wafer or bare die) containing the fabricated devices is flipped by means of flip‐chip bonding or temporary bonding with frontside protection with photoresist. Then, the back side of the substrate is etched using DRIE to reduce its thickness.^[^ ^393^ ^]^ Individual devices such as FinFETs, sensors, capacitors, charge‐trapping memory devices, and microbattery have been successfully flexed using this technique (see Figure 6b).^[^ ^383^, ^386^, ^393^, ^394^, ^395^, ^396^ ^]^ Although this approach sacrifices a bulk of the substrate during the flexing step, it is more advantageous because it has a lower integration complexity (i.e., fewer steps) and there is no area loss due to trench formation.

By utilizing this approach, Ghoneim et al. reported a flexible nonvolatile memory based on PZT ferroelectric capacitors.^[^ ^396^ ^]^ The device exhibits a minimum bending radius of 5 mm and remains stable even after 1300 bending cycles at this radius. Similarly, Das et al. demonstrated flexible silicon‐based charge‐trapping memory devices.^[^ ^395^ ^]^ When bent at a bending radius of 20 mm, the flexible memory devices show minimal deviation in threshold voltage in comparison to those of the rigid counterparts. Moreover, in conjunction with heterogeneous integration, it is possible to fabricate compact fully standalone flexible electronic systems. Shaikh et al. reported a modular heterogeneous integration scheme inspired by LEGO (see Figure 6c).^[^ ^29^ ^]^ Conductive LEGO teeth are fabricated on the contact pads of a bare die, and the dies are assembled on a flexible substrate with interconnects and sockets specific to each die type. Sequential back‐etch is done to flex the assembled bare dies exhibiting a minimum bending radius of 0.5 mm as shown in Figure 6d. Combining this with multiple sensors, it is possible to fabricate a fully flexible multimodal sensory platform as introduced in ^[^ ^384^ ^]^ (see Figure 6e). The underwater tagging device features pressure, salinity, and temperature sensors as well as a bare‐die MCU and a solid‐state battery (Figures 6f,g). The device can be gently mounted on the surface of a marine species and thus does not disturb the animal's behavioral pattern. and is operational in a water depth of up to 2 km. It is possible to incorporate a BLE bare die and an antenna in the system to equip the system with wireless communication capabilities as well (see Figure 6i).^[^ ^385^ ^]^ Potential battery capacity issues can also be mitigated by integrating multiple solid‐state batteries in parallel as reported by Kutbee et al. as shown in Figure 6j.^[^ ^386^ ^]^ An example of a system addressing the potential issues discussed thus far is the device reported by Shaikh et al., which incorporates a bare‐die MCU, BLE, and solid‐state batteries.^[^ ^27^ ^]^

## 4 Conclusion and Future Outlook

In this paper, the recent progress in flexible electronics is reviewed from the integration standpoint. The essential building blocks of a fully standalone flexible electronic system include sensors, front‐end circuitry, data management, power management, and optionally actuation. Because the vast majority of flexible devices are used in wearable and healthcare applications, there is a myriad of flexible sensors reported in the community: temperature, pressure, mechanical strain, humidity, pH level, biopotential, chemical, etc. Such sensors provide invaluable information about the physiological status of the user^[^ ^10^, ^305^, ^355^, ^364^ ^]^ or the environment^[^ ^381^, ^384^ ^]^ in various forms: resistance, capacitance, electrical potential, and current. For instance, temperature is an important physiological indicator of the user's health or wound recovery process.^[^ ^37^, ^252^ ^]^ Physically conformal flexible sensors can be deployed to continuously monitor subtle changes in their physiological conditions and inform the user of their conditions. In environmental sensing, compliant, lightweight IoT devices can be deployed to enable non‐invasive remote continuous monitoring of the environment.

The sensor output data must be preconditioned using appropriate front‐end circuitry, which converts them into an electrical voltage (if necessary) and amplifies the signal. Then, the signal is digitized by an ADC before being interfaced to the MCU for further processing. The preconditioned raw data now can be interpreted by the MCU to infer the change in the sensing parameters. The processed data can be wirelessly transmitted to a nearby smart device (e.g., tablet or smartphone) via BLE to display the physiological/environmental information. If there is an on‐system actuator (e.g., for therapy purposes), an algorithm programmed into the MCU can autonomously control it. If the sensing parameter extraction demands heavy computation that cannot be handled by the MCU, the preconditioned raw data can be directly wirelessly transmitted to a nearby smart device; this approach is typically used if the raw data must be further filtered and fed into a neural network for regression or classification tasks.

There are multiple reported strategies to integrate all the building blocks above to construct fully standalone flexible electronic systems. Organic electronic systems, despite their unmatched flexibility and stretchability, their performance is typically limited, stemming from the low mobile charge carrier mobility values. Furthermore, their integration density is lower compared to that of the state‐of‐the‐art CMOS technology. Flexible hybrid electronic systems utilize state‐of‐the‐art CMOS components for their circuits. However, the use of rigid packaged components presents an inherent limit in flexibility.

As an alternative to both, transformational electronic systems, where bare‐die components with small dimensions are utilized to construct necessary circuits for data and power management. Compact integration of bare‐die components can increase the integration density, thereby reducing the overall system footprint. Moreover, by using the sequential back‐etch technique, it is possible to thin down the bare dies and thus significantly enhance their flexibility while retaining their functionality. Furthermore, as the semiconductor industry is heading toward 3D heterogenous integration of dissimilar chiplets to further increase the integration density, it is natural to adopt similar approaches to make flexible electronic systems even more compact. The 3D heterogeneous integration of state‐of‐the‐art CMOS bare dies, utilizing sequential back‐etch techniques and advanced on‐site data analysis algorithms, will enable the next generation of flexible electronic systems to achieve ultra‐compact sizes, high flexibility, exceptional data‐processing capabilities, and superior energy efficiency.

## Conflict of Interest

The authors declare no conflict of interest.
